# Children’s Early Spontaneous Comparisons Predict Later Analogical Reasoning Skills: An Investigation of Parental Influence

**DOI:** 10.1162/opmi_a_00093

**Published:** 2023-07-28

**Authors:** Catriona Silvey, Dedre Gentner, Lindsey Engle Richland, Susan Goldin-Meadow

**Affiliations:** Division of Psychology and Language Sciences, University College London, London, UK; Department of Psychology, Northwestern University, Evanston, IL, USA; School of Education, University of California, Irvine, Irvine, CA, USA; Department of Psychology, University of Chicago, Chicago, IL, USA; Department of Comparative Human Development, University of Chicago, Chicago, IL, USA

**Keywords:** comparison, analogical reasoning, cognitive development

## Abstract

Laboratory studies have demonstrated beneficial effects of making comparisons on children’s analogical reasoning skills. We extend this finding to an observational dataset comprising 42 children. The prevalence of specific comparisons, which identify a feature of similarity or difference, in children’s spontaneous speech from 14–58 months is associated with higher scores in tests of verbal and non-verbal analogy in 6th grade. We test two pre-registered hypotheses about how parents influence children’s production of specific comparisons: 1) via modelling, where parents produce specific comparisons during the sessions prior to child onset of this behaviour; 2) via responsiveness, where parents respond to their children’s earliest specific comparisons in variably engaged ways. We do not find that parent modelling or responsiveness predicts children’s production of specific comparisons. However, one of our pre-registered control analyses suggests that parents’ global comparisons—comparisons that do not identify a specific feature of similarity or difference—may bootstrap children’s later production of specific comparisons, controlling for parent IQ. We present exploratory analyses following up on this finding and suggest avenues for future confirmatory research. The results illuminate a potential route by which parents’ behaviour may influence children’s early spontaneous comparisons and potentially their later analogical reasoning skills.

## INTRODUCTION

Comparison is the process of noticing the similarities and differences between two objects or events. Previous work has argued that the process of comparison is crucial in the development of children’s word learning, categorisation, and analogical reasoning skills (Gentner, 2010; Gentner & Namy, 2006; Gentner et al., 2011; Namy & Gentner, 2002; Richland & Simms, 2015). Comparison is argued to be an effective learning tool because it promotes structural alignment: the mapping of two representations in a way that promotes the recognition of relational commonalities and alignable differences (Goldwater & Gentner, 2015; Markman & Gentner, 1993). Indeed, experimental work has shown that inviting children to compare exemplars helps them move beyond overall or global similarity to more specific kinds of similarity, including similarity based on the relational commonalities that underlie analogical reasoning (e.g., Christie & Gentner, 2010; Gentner, 2010; Gentner et al., 2016; Haryu et al., 2011; Loewenstein & Gentner, 2001; Simms & Richland, 2019).

Despite this rich tradition of experimental work showing the benefits of elicited comparison for children’s relational insight, it is currently less clear how this relates to children’s spontaneous comparison in naturalistic contexts. Children produce comparative utterances from early in their language development, spontaneously generating metaphors from the age of around 2 (Winner, 1979). Özçalışkan et al. (2009) examined the development of children’s spontaneous comparisons from the age of 14 to 34 months during naturalistic interactions with their primary caregiver in the home (an earlier iteration of the dataset analysed in the current study). The researchers found a developmental change in the types of comparisons children produced over time. Children’s earliest comparisons tended to be between objects that were similar to each other in many features. However, the acquisition of the word ‘like’ (from around 26 months) was associated with an increase in the number of comparisons between objects that only shared a single feature. In what follows, we define comparisons that pick out a single feature of similarity or difference (e.g., a child commenting that a piece of candy is the same colour as his shirt) as *specific* comparisons, and comparisons that do not pick out a single feature of similarity or difference (e.g., a child commenting that her balloon is like her sibling’s balloon) as *global* comparisons.^1^ Theoretical work has argued that global similarity is developmentally prior to single-feature similarity (Smith, 1989), based on children’s behaviour in sorting tasks, and on the fact that words referring to object categories are learned before words referring to features such as colour. Özçalışkan et al.’s finding that the earliest comparisons tend to be global reinforces this point, and suggests that specific comparisons signal a child attaining a more mature stage in their development of this cognitive capacity. While not necessarily relational in themselves, specific comparisons thus constitute an early stepping-stone on the way to the relational kinds of similarity that underlie analogical reasoning (Gentner, 1988; Gentner & Rattermann, 1991). In Özçalışkan et al.’s study, children varied in the extent to which they produced specific comparisons; however, the authors did not investigate the relationship between this variation and later analogical reasoning outcomes.

Further work on the same dataset investigated children’s comparisons as one aspect of the development of higher-order thinking (Frausel et al., 2020). Modelling the growth of higher-order thinking in children’s spontaneous speech from age 14 months to 58 months, Frausel and colleagues found that children began producing utterances that demonstrate higher-order thinking around the age of 23–27 months. The researchers also discovered a developmental progression, in that the onset of less complex forms of thinking (‘surface’) preceded the onset of more sophisticated, abstract forms (‘structure’). Children varied substantially in the amount of higher-order thinking they expressed and their growth rate over time; crucially, this variation predicted children’s outcomes in later standardised tests of higher-order thinking skills, including tests of verbal and non-verbal analogical reasoning. However, the study did not examine comparisons in isolation, but considered them together with other, more frequently observed types of higher-order thinking, such as inference. As such, this study does not specifically clarify the relationship between early spontaneous comparisons and children’s later analogical reasoning outcomes.

Taken together, these previous studies suggest that the prevalence of specific comparisons in children’s early speech could potentially be an index of their later analogical reasoning skill. Indeed, previous work with a sample of 24 children from the same dataset revealed that the number of specific comparisons children produced in spontaneous talk between the ages of 14 and 58 months related strongly to their performance in tests of analogical reasoning given much later, in 6th grade (Silvey et al., 2017). One of the aims of this paper is to replicate and extend this result in a larger sample of children. Another is to ask whether parents’ behaviour influences the prevalence of specific comparisons in children’s early spontaneous speech. Parents provide examples of comparisons in speech input to the child; they also act as interaction partners, giving more or less engaged responses to comparisons the child produces. Previous work looking at children’s language development in general has found that parents’ input (e.g., Silvey et al., 2021) and responsiveness to their children (e.g., Tamis-LeMonda et al., 2001) both have a substantial influence on children’s subsequent language skill. However, previous work on the current dataset has not investigated either the comparisons parents produce, or the ways parents respond to their children’s comparisons, leaving the question of parents’ potential influence on their children’s comparisons as yet unaddressed.

To summarise, this paper has three main aims. Aim 1 is to describe the spontaneous comparisons children and their parents make in the context of naturalistic interactions in the home when children are aged between 14 and 58 months. Our characterisation of children’s comparisons builds on work by Özçalışkan and colleagues (2009), Silvey and colleagues (2017), and Frausel and colleagues (2020). Following these researchers, we focus on the words children used to express comparisons, whether comparisons were global or specific, and whether comparisons involved objects from the same or different superordinate categories. We further investigate which features children’s specific comparisons tend to single out, to provide insight into the naturalistic contexts that most readily give rise to comparisons. Finally, we code whether children’s comparisons highlight similarity or difference. Previous work has generally focused on similarity, but the recognition of alignable differences is also an important consequence of structural alignment. We then compare parents’ comparisons to their children’s in terms of these characteristics. To our knowledge, the characteristics of parents’ comparisons in speech to children during this period have not previously been systematically described.

Aim 2 is to extend Silvey and colleagues’ (2017)’s finding, that the prevalence of specific comparisons in children’s speech from 14–58 months predicts their performance in tests of analogical reasoning administered in 6th grade, to a larger sample with more robust analyses. Specifically, we increase the sample of children from 24 to 42 and run two novel control analyses investigating whether the relationship between specific comparisons and analogical reasoning can be alternatively explained by children’s production of comparisons in general, or children’s overall language competence.

Aim 3 is to test two hypotheses about how parents’ behaviour might influence their children’s production of specific comparisons. The *modelling* hypothesis posits that children of parents who produce more specific comparisons in early sessions will go on to produce more specific comparisons themselves. That is, a parent who frequently comments on objects in the child’s environment being, for example, the same colour, or bigger or smaller than each other, is providing crucial input that supports the child in going on to produce these kinds of comparisons themselves. The *responsiveness* hypothesis posits that children of parents who respond in a high-engagement way to their children’s earliest specific comparisons will go on to produce more specific comparisons in subsequent sessions. For example, if a child comments that a toy is the same colour as her shoes, and the parent engages with the child’s comparison by asking an elaborating question (‘and what colour is that?’), we might expect that child to make more of these specific comparisons in the future. Aim 3 is novel to the current paper; the hypotheses and analysis plan were preregistered and can be found on the Open Science Framework at https://osf.io/gaq97.

## AIM 1: TO DESCRIBE CHILDREN’S (A) AND PARENTS’ (B) SPONTANEOUS COMPARISONS

### Aim 1 Method

#### Data.

Data were taken from a longitudinal study of language development (Goldin-Meadow et al., 2014) approved by the Institutional Review Board of the University of Chicago. Participants were 42 children (18 girls, 25 first-borns) and their primary caregivers. This sample included the 24 participants originally analysed in Silvey et al. (2017) and 18 additional participants. Children were visited at home every 4 months from age 14 to 58 months, and videotaped for 90 minutes interacting with their parents. Families were instructed to carry on with their normal daily activities; as such, the interactional content of the sessions varied naturally within and between families (e.g., mealtimes, playing with toys, book reading, etc.). All parent and child speech was transcribed at the utterance level. The spontaneous comparison data are drawn from these observational sessions. The 42 children included in the current study were randomly selected from the original sample of 64 under two constraints: 1) to ensure complete data from the 12 observational sessions and 2) to preserve, as far as possible, the diversity of the original sample, which was representative of the greater Chicago area in terms of race, ethnicity, and income.

#### Comparison Coding.

Comparisons were first identified from the transcripts of the observational sessions. This was done as part of a larger project investigating the development of higher-order thinking (Frausel et al., 2020). We then coded these comparisons more finely, with reference both to the transcript and to the original video of the session. The full coding scheme is provided in Text S1 of the Supplementary Material. Except where otherwise stated, we coded both parents’ and children’s comparisons for each of the variables listed below. Reliability was assessed by having two members of the research team code all the comparisons produced by 5 randomly selected parent-child pairs. Reliability was assessed for parent and child comparisons separately, and was quantified via Cohen’s kappa (Cohen, 1960). Agreement in all cases was substantial, with all kappas greater than .6; specific values for each variable are given below. Further details on coding, reliability, and examples of disagreements and how they were resolved are given in Text S2 of the Supplementary Material.

##### *Word Category*.

We coded the word(s) that made the utterance a comparison. For example, in the utterance ‘I’m a silly one like you’^2^, the word is ‘like’. We classified these words into the following categories: like (the words ‘like’ and ‘alike’), same/different (the words ‘same’ and ‘different’), too (used either in contexts like ‘too big’ or contexts like ‘I’m running too’), comparative/superlative (e.g., ‘bigger’, ‘best’), match (e.g., ‘this one matches this one’), and other. Reliability was .82 for parents’ comparisons and .78 for children’s.

##### *Similarity vs. Difference*.

We coded whether each comparison expressed similarity (e.g., ‘go like a tiger’) or difference (e.g., ‘I’m taller than everybody!’) Reliability was .76 for parents and .70 for children.

##### *Global vs. Specific*.

We coded each comparison for whether it expressed global similarity/difference (e.g., ‘I have dolls just like yours’) or specific similarity/difference (e.g., ‘red like the tomato’), picking out a particular feature of similarity or difference. Reliability was .72 for parents and .71 for children.

##### *Feature Specified*.

For specific comparisons, we further coded what feature was specified. Features were classified into 6 categories: Spatial (e.g., size, shape, distance, speed), Sensory (e.g., colour, weight, taste, smell), Evaluative (e.g., goodness, prettiness, badness), Physiological (e.g., strength, tiredness), Preference (e.g., liking something better than something else^3^), and Other. Reliability was .70 for parents and .72 for children. Features were further classified as Perceptual (based on a readily perceptible attribute, e.g., colour, size) or Non-Perceptual (based on a more abstract feature, e.g., preference, goodness). Reliability was .71 for both parents and children.

##### *Within or Between-category*.

Comparisons were coded for whether the objects involved in the comparison were from the same or different superordinate categories. Objects within the same category tend to share more overall similarity, making within-category comparisons potentially more accessible for children from a younger age. Superordinate categories were taken from Özçalışkan et al. (2009), with additions to accommodate new data (in italics): people, animals, body parts, vehicles, clothing, furniture, appliances, kitchen utensils, tools, musical instruments, food, plants, activity toys, *decorations*/*crafts*, *words*/*letters*, and *shapes*. Reliability was .62 for both parents and children.

Aim 1 is to describe the overall prevalence of different types of comparisons in parent and child speech and how the comparisons change between child age 14 and 58 months. We also investigate whether some types of comparison are generally produced before other types, either by children (suggesting developmental ordering) or by parents (suggesting tailoring of input to children’s developmental level). Finally, we investigate whether some types of comparison are interdependent (e.g., whether within-category comparisons tend to be disproportionately specific rather than global).

### Aim 1 Results

#### Aim 1a: To Describe Children’s Spontaneous Comparisons

##### *Onset and Prevalence of Comparisons Over Time*.

**Figure 1.**
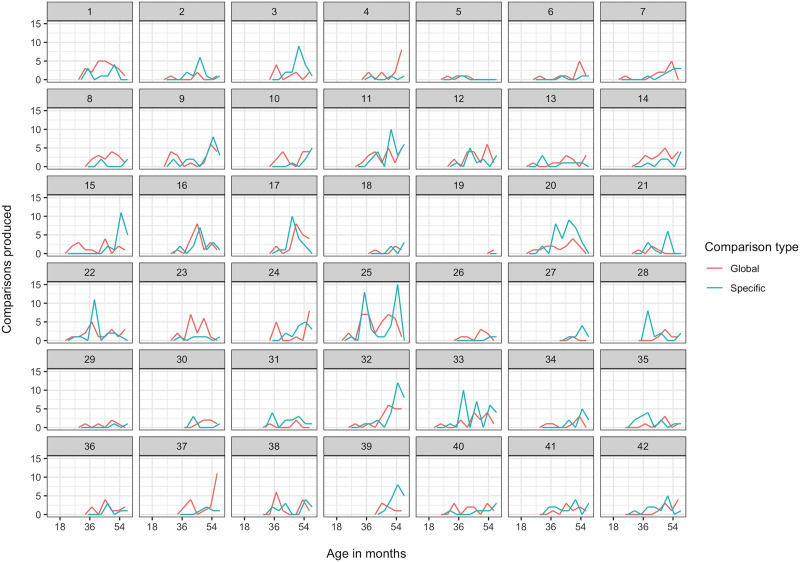
Number of global (red) and specific (blue) comparisons produced by each child (separate plots) over the period from 14 to 58 months (*x* axis).

We define onset as the first session in which the child produces at least one comparison, provided the child also produces at least one comparison during the immediately following session.^4^ Median onset was session 7, when children were 38 months old. Earliest onset was session 4, when children were 26 months old, and the latest measurable onset was session 10, when children were 50 months old. Comparisons ranged from 0% to 3.7% of a child’s utterances in a given session. Figure 1 shows the absolute number of global and specific comparisons each child produced over the 12 sessions. Since no child produced a comparison before session 4 (26 months), the remaining figures in this section show only sessions 4–12.

##### *Global and Specific Comparisons*.

Global and specific comparisons were equally frequent on average (506 global vs. 509 specific). However, as Figure 1 suggests, children tended to start producing global comparisons either before or at the same time as specific comparisons. Of the 41 children who produced both, 22 produced a global comparison before they produced a specific comparison, 11 produced both during the same session, and 8 produced a specific comparison first. This tendency for global comparisons to precede specific comparisons, rather than vice versa, differed significantly from chance: *χ*^2^(1) = 6.5, *p* = .011.^5^

##### *Features Specified*.

The most frequently specified features were spatial or sensory; together, these accounted for 67% of the specific comparisons the children expressed. Table 1 shows overall counts and percentages.

**Table 1.** Frequency of feature categories specified in children’s comparisons.

**Feature category**	**Number of uses**	**Percent of specific comparisons**
Spatial	231	45%
Sensory	113	22%
Evaluative	101	20%
Other	50	10%
Physiological	11	2%
Preference	3	1%

Perceptual features were specified around twice as frequently as non-perceptual features: 344 (68%) versus 165 (32%). Children tended to specify perceptual features earlier than they specified non-perceptual features: of the 35 children who specified both, 23 specified perceptual features before they specified non-perceptual features, 6 specified both in the same session, and 6 specified non-perceptual features earlier. This tendency for perceptual features to be specified earlier differed significantly from chance: *χ*^2^(1) = 9.97, *p* = .002.

##### *Comparison Words*.

The most frequently used comparison word was ‘like’, followed by ‘too’, ‘same’, ‘bigger’, ‘different’, and ‘better’. Together, these words accounted for 75% of the comparisons the children expressed. Table 2 shows counts and percentages for each word category.

**Table 2.** Frequency of comparison word categories.

**Word category**	**Number of uses**	**Percent of all comparisons**
like	411	41%
comparative/superlative	244	24%
too	156	15%
same/different	98	10%
other	72	7%
match	34	3%

**Figure 2.**
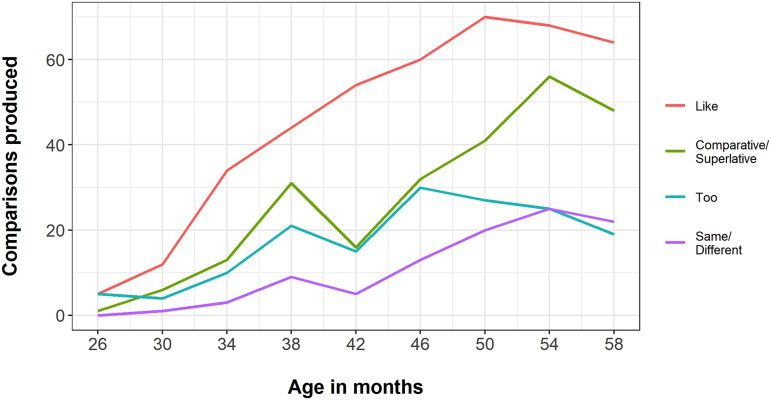
Overall frequency summed over children of word categories ‘like’ (red), ‘comparative/superlative’ (green), ‘too’ (blue) and ‘same/different’ (purple) over the period from 26 to 58 months (*x* axis).

Figure 2 shows the overall frequencies of the four most prevalent word categories over time. ‘Like’ was the first word category to reliably emerge. While ‘like’ and comparatives/superlatives were overall the most frequent, all word categories generally showed an increase in use across sessions.

Building on Özçalışkan and colleagues’ (2009) finding that the emergence of ‘like’ coincided with children beginning to make comparisons between objects that only shared one feature, we also examined the relationship between the use of the word ‘like’ and whether a comparison was global or specific. ‘Like’ was more prevalent in children’s global comparisons (appearing in 61% of global comparisons) than in their specific comparisons (appearing in 20% of specific comparisons). While this may seem to contradict Özçalışkan and colleagues, it is important to bear in mind that, as explained in footnote 1, we defined specific comparisons in terms of the *language* the child used, not the number of features shared between the compared objects.^6^ Therefore, some comparisons we classify as global may be single-feature comparisons under Özçalışkan and colleagues’ definition, and some comparisons they classified as many-feature may have been specific based on the language the children used. However, our data do suggest that the word ‘like’ offers children a way into comparison by enabling the majority of their global comparisons.

##### *Expressing Similarity and Difference*.

**Figure 3.**
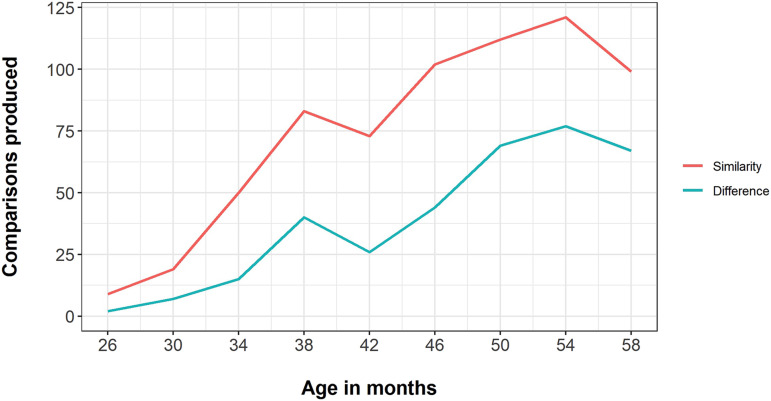
Overall frequency summed over children of comparisons that expressed similarity (red) and difference (blue) over the period from 26 to 58 months (*x* axis).

Comparisons expressing similarity were almost twice as frequent as comparisons expressing difference (668 vs. 347). Figure 3 shows the overall frequency of similarity and difference comparisons over time. As Figure 3 suggests, children generally remarked on similarities before they remarked on differences. Of the 40 children who produced both types of comparison, 30 produced a similarity comparison before they produced a difference comparison, 3 produced a difference comparison before they produced a similarity comparison, and 7 produced both during the same session. This tendency for similarities to precede differences was significantly different from chance: *χ*^2^(1) = 22.1, *p* < .001.

##### *Within- and Between-category Comparisons*.

Comparisons between objects in the same category (or between events involving objects in the same category) were around three times more frequent than between-category comparisons (773 versus 240). As predicted, children generally made within-category comparisons before they made between-category comparisons: of the 39 children who produced both, 26 produced within-category comparisons earlier than between-category comparisons, 7 produced both during the same session, and 6 produced between-category comparisons first. This tendency for within-category comparisons to come first differed significantly from chance: *χ*^2^(1) = 12.5, *p* < .001.

We also looked at how within- and between-category comparisons interacted with two other variables: whether comparisons were global or specific, and whether they highlighted a similarity or a difference. 395 (51%) of children’s within-category comparisons were specific, compared to 114 (48%) of between-category comparisons. This was not significantly different from chance: *χ*^2^(1) = 0.8, *p* = .368. 463 (60%) of children’s within-category comparisons highlighted a similarity, compared to 203 (85%) of between-category comparisons. This differed significantly from chance: *χ*^2^(1) = 48.5, *p* < .001.

#### Aim 1b: To Describe Parents’ Spontaneous Comparisons

##### *Onset and Prevalence of Comparisons Over Time*.

**Figure 4.**
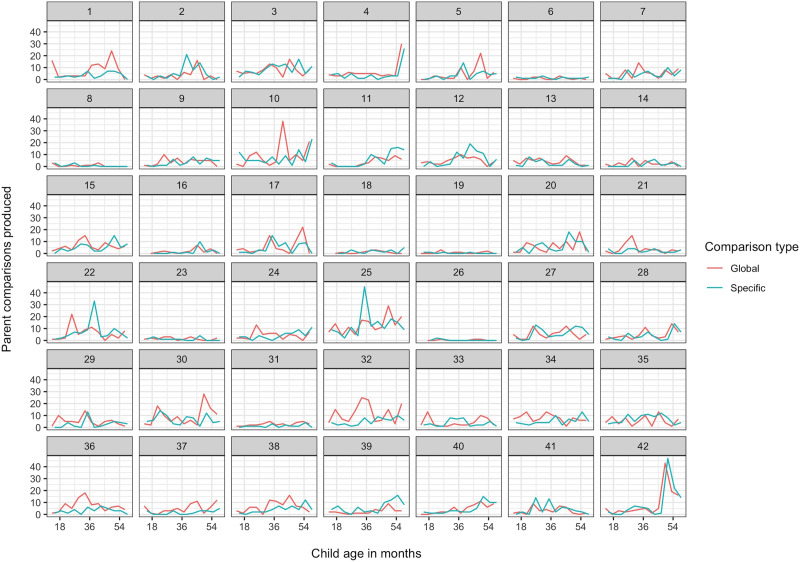
Number of global (red) and specific (blue) comparisons produced by each parent (separate plots) over the period from child age 14 to 58 months (*x* axis).

The majority of parents (38 out of 42) produced comparisons from the first observation session, when their child was 14 months old. Of the remaining 4 parents, one produced their first comparison in session 2 (18 months), and three in session 3 (22 months). Comparisons ranged from 0% to 6.2% of parents’ utterances in a given session. Figure 4 shows the absolute number of global and specific comparisons each parent produced over the 12 sessions.

For parents, we report only a subset of the analyses we report for children. In particular, we do not report the word categories used by parents, since these were similar overall and over time to the patterns we saw in children.

##### *Global and Specific Comparisons*.

Global comparisons were slightly more frequent overall in parents’ speech than specific comparisons (2492 global vs. 2154 specific). As Figure 4 suggests, the majority of parents began producing both types of comparison around the same time: 8 parents produced a global comparison before they produced a specific comparison, 30 produced both during the same session, and 4 produced a specific comparison first. A chi square test found no significant ordering tendency: *χ*^2^(1) = 1.33, *p* = .248. This contrasts with the significant tendency for children to produce global comparisons first, suggesting that, on the whole, parents were not tailoring the specificity of the comparisons they made to children’s developmental stage.

As for children, we were also interested in how parents’ use of ‘like’ varied across global and specific comparisons. 69% of parents’ global comparisons used the word ‘like’, whereas only 14% of parents’ specific comparisons used this word. In parents’ speech, ‘like’ was more characteristically associated with global comparisons than in children’s speech.

##### *Features Specified*.

The features most frequently specified by parents were spatial or evaluative; together, these accounted for 57% of the specific comparisons the parents expressed. Table 3 shows overall counts and percentages. Overall, the parents’ proportions are similar to their children’s (Table 1); parents produced proportionally fewer spatial and sensory comparisons and more evaluative comparisons. They also produced proportionally more comparisons that fell into the ‘other’ category, suggesting that parents’ specific comparisons were more diverse than their children’s in topic overall.

**Table 3.** Frequency of feature categories specified in parents’ comparisons.

**Feature category**	**Number of uses**	**Percent of specific comparisons**
Spatial	763	35%
Evaluative	480	22%
Sensory	434	20%
Other	373	17%
Preference	54	3%
Physiological	50	2%

As this pattern might suggest, parents also specified proportionally more non-perceptual features than children: 1189 perceptual (55%) versus 965 non-perceptual (45%). The majority of parents began producing both of these around the same time: 10 parents specified a perceptual feature before they specified a non-perceptual feature, 13 did the reverse, and 19 specified both in the same session. A chi square test found no significant ordering tendency: *χ*^2^(1) = 0.39, *p* = .532. As above, this suggests that parents were, on the whole, not tailoring the type of comparisons they produced to children’s developmental stage. This was also the case for parents’ production of comparisons that pointed out similarity versus difference: 11 produced a similarity comparison first, 4 a difference comparison first, and 27 produced both in the same session. A chi square test found no significant ordering tendency: *χ*^2^(1) = 3.27, *p* = .071. The prevalence of similarity versus difference in parents’ comparisons was similar to their children’s: 2884 similarity comparisons (62%) versus 1762 difference comparisons (38%).

##### *Within- and Between-category Comparisons*.

Parents were similar to their children in the relative frequency of comparisons that involved objects in the same category: 3582 (77%) within-category, compared to 1058 (23%) between-category. 20 parents made within-category comparisons before they made between-category comparisons, 2 did the reverse, and 19 did both in the same session. This tendency to produce within-category comparisons first was significant: *χ*^2^(1) = 14.73, *p* < .001. However, as the numbers show, parents were evenly split on whether they made this ordering distinction or made no distinction. One possibility is that a subset of parents was ordering these types of comparisons in a way designed to be helpful for their children, as predicted by the theory of progressive alignment (Gentner et al., 2011; Kotovsky & Gentner, 1996). We investigate this possibility in the exploratory analyses.

As we did for children, we also examined how parents’ within- and between-category comparisons interacted with two other variables: whether comparisons were global or specific, and whether they highlighted a similarity or a difference. 1838 (51%) of parents’ within-category comparisons were specific, compared to 314 (30%) of between-category comparisons. This differed significantly from chance: *χ*^2^(1) = 152.84, *p* < .001. Unlike their children, parents appeared to specify features of similarity or difference more often when the objects or events compared were in the same category. For parents, 1965 (55%) of within-category comparisons highlighted a similarity, compared to 914 (86%) of between-category comparisons. Similar to the pattern we observed in children, this differed significantly from chance: *χ*^2^(1) = 343.51, *p* < .001.

Table 4 summarises the patterns we describe in parents’ and children’s comparisons from child ages 14–58 months.

**Table 4.** Patterns observed in children’s and their parents’ comparisons from child age 14–58 months.

		**Children**	**Parents**
**Global vs. specific**	**Global (%)**	50	54
	**Specific (%)**	50	46
	**Ordering trend**	Global -> Specific	Simultaneous
**Features specified**	**Most frequent categories**	Spatial, Sensory (67% of total)	Spatial, Evaluative (57% of total)
	**Perceptual (%)**	68	55
	**Non-perceptual (%)**	32	45
	**Ordering trend**	Perceptual -> Non-perceptual	Simultaneous
**Similarity vs. difference**	**Similarity (%)**	66	62
	**Difference (%)**	34	38
	**Ordering trend**	Similarity -> Difference	Simultaneous
**Within vs. between category**	**Within (%)**	76	77
	**Between (%)**	24	23
	**Ordering trend**	Within -> Between	Within -> Between

### Aim 1 Discussion

We described the comparisons produced in naturalistic speech by parents and children in the home between child age 14 and 58 months. Children started producing comparisons at a median age of 38 months, and no child produced a comparison before session 4 (age 26 months). Following onset, comparisons became an infrequent but robust feature of children’s speech. Children generally produced global comparisons before specific comparisons, similarity comparisons before difference comparisons, and within-category comparisons before between-category comparisons. When children began to produce specific comparisons, they generally commented on perceptual features (such as colour and size) before they commented on non-perceptual features (such as goodness or preference). The majority of children’s specific comparisons talked about spatial or sensory features. The key words underlying children’s use of comparisons in their speech were ‘like’, comparative and superlative adjectives, ‘too’, and the words ‘same’ and ‘different’. Children were equally likely to produce a specific comparison about two objects from the same category as two objects from different categories; however, when expressing a comparison between two objects from different categories, children were more likely to talk about their similarities than their differences. These findings about the kinds of comparisons children tend to express in their early spontaneous speech can help inform the design of materials for experimental studies, ensuring they are ecologically valid to children’s experiences in the home.

Parents mostly began producing comparisons from the beginning of our observation period, when their children were 14 months old. By contrast with their children, parents did not display significant ordering tendencies for the different types of comparison. The only exception was within- and between-category comparisons, where half of parents produced within-category comparisons first (in accordance with the theory of progressive alignment); the other half of parents did not make an ordering distinction.

Parents’ specific comparisons mostly talked about spatial or evaluative features, and the range of features they specified was more diverse than children’s, as might be expected given children’s relatively narrower linguistic and overall experience. Parents also differed from their children in that they were less likely to produce a specific comparison about two objects from different categories than they were about two objects from the same category. Like their children, they tended to talk more about similarities than differences between objects from different categories.

## AIM 2: TO INVESTIGATE THE RELATIONSHIP BETWEEN CHILDREN’S EARLY SPECIFIC COMPARISONS AND LATER ANALOGICAL REASONING

### Aim 2 Method

#### Data.

Measures of children’s production of spontaneous comparisons and other speech were taken from the same longitudinal dataset of 42 children described under Aim 1. Household income and primary caregiver years of education were collected via report at the first visit, when children were 14 months old. Later, when the children were in school, they were administered tests of verbal and non-verbal analogical reasoning.

#### Variables

##### *Annual Household Income*.

Income was coded as one of 6 categories: between $0 and $14,999 (3 families in our sample); between $15,000 and $34,999 (6 families); between $35,000 and $49,999 (6 families); between $50,000 and $74,999 (10 families); between $75,000 and $99,999 (7 families); and $100,000 or above (10 families). The midpoint of each category in thousands of dollars was assigned as the value of household income, except for the highest category, which was assigned a value of 100 (i.e., $100,000).

##### *Parent Education*.

Education was coded as one of five categories: some high school (10 years – 2 parents in our sample), high school/GED (12 years – 3 parents in our sample), some college or trade school (14 years – 5 parents in our sample), bachelor’s degree (16 years – 17 parents in our sample), and advanced degree (18 years – 15 parents in our sample).

##### *Composite Measure of Socio-economic Status*.

Household income and parent education were converted to *z*-scores and then averaged to produce a composite measure of socio-economic status (SES).

The remaining variables are the same as those reported in Silvey et al. (2017), with additional control analyses and other minor differences noted below.

##### *Specific Comparisons Children Produced During Sessions 1–12*.

The total number of specific comparisons (see above) that each child produced during the 12 observational sessions from 14-58 months is our predictor of interest. Silvey et al. (2017) neglected to account for the fact that the distribution of this variable is skewed; in our analyses, we account for this by taking the natural logarithm of this variable before entering it in our models.^7^ Note that since no child produced a comparison before session 4, in practice this count was taken from sessions 4–12.

##### *Global Comparisons Children Produced During Sessions 1–12*.

The total number of global comparisons (see above) that each child produced during the 12 observational sessions from 14-58 months is the predictor in our first control analysis. As for specific comparisons, the distribution of this variable is skewed; we therefore take the natural logarithm of this variable before entering it in our models. Note that since no child produced a comparison before session 4, in practice this count was taken from sessions 4–12.

##### *Number of Utterances Children Produced During Sessions 1–12*.

The total number of utterances each child produced during the 12 observational sessions from 14–58 months is the predictor in our second control analysis.

##### *Children’s Vocabulary*.

The Peabody Picture Vocabulary Test (PPVT; Dunn & Dunn, 1997), a widely used test of receptive vocabulary, was administered during the penultimate observational session at 54 months.

##### *Children’s Scores on Analogical Reasoning Tests*.

We administered two tests of analogical reasoning when the children were in 6th grade (aged around 11 years): the Verbal Analogies subtest of the Woodcock-Johnson Tests of Cognitive Abilities (WJ-VA; Woodcock et al., 2001), and a non-verbal test, Raven’s Progressive Matrices (Raven et al., 2003). The WJ-VA is an orally administered test that consists of sets of paired items. The participant has to fill in the missing item by abstracting the relation that holds between the first pair. For example, the participant is given the prompt ‘mother is to father, as sister is to …’, and expected to fill in the missing term ‘brother’. Raven’s Progressive Matrices are a series of geometric analogy problems. The participant is presented with a matrix that has one entry missing and must select the correct entry from an array of 6–8 choices.

Aim 2 is to investigate the relationship between children’s early specific comparisons and their later analogical reasoning skills. To this end, we report three analyses: 1) our main analysis replicating and extending Silvey et al. (2017), predicting children’s scores in verbal and non-verbal analogy tests from the log number of specific comparisons the child produced during the twelve observational sessions; 2) our first control analysis, predicting child analogy scores from the log number of *global* comparisons the child produced during the twelve observational sessions; 3) our second control analysis, predicting child analogy scores from the total number of utterances the child produced during the twelve observational sessions. The first control analysis accounts for the possibility that what looks like an association with specific comparisons may, in fact, be an association with comparisons in general. The second control analysis accounts for the possibility that what looks like an association with specific comparisons may, in fact, be an association with a child’s overall talkativeness. We run these models separately, rather than entering all three predictors into the same model, because we are not trying to test which of these three predictors explains the outcome over and above the others when considered simultaneously. Rather, we are testing distinct hypotheses about whether a more general construct, such as overall comparisons or overall talkativeness, predicts our outcome as well as our theoretically motivated predictor, specific comparisons. If we find a significant relationship in our main analysis but not in either of our control analyses, this finding would strengthen the claim that it is specific comparisons *per se* that matter. Following Silvey et al. (2017), we include child PPVT score in each of our outcome models to control for children’s language proficiency, which could influence both the production of specific comparisons and later analogical reasoning. Finally, we run an additional analysis investigating the relationship between family socio-economic status, children’s production of specific comparisons, and children’s scores in analogical reasoning tests.

### Aim 2 Results

#### *Main Analysis*.

**Figure 5.**
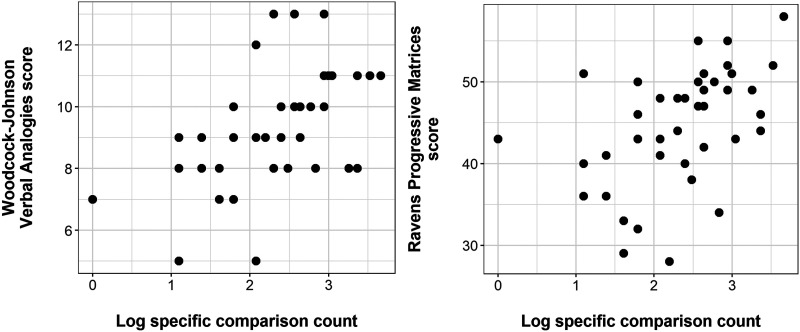
Scatterplots showing the relationship between our key predictor, log specific comparison count (*x* axis) and analogical reasoning outcomes: score in Woodcock-Johnson Verbal Analogies (*y* axis, left panel) and Raven’s Progressive Matrices (*y* axis, right panel).

Our main analysis found that log specific comparison count was a significant predictor of score in Woodcock-Johnson Verbal Analogies: *b* = 1.29, *t* = 3.86, *p* < .001. This corresponded to a standardised effect size of 0.52, a medium effect. Adjusted *R*^2^ for the model was .25, suggesting that this predictor explained around a quarter of the variance in verbal analogy scores. We also found that log specific comparison count was a significant predictor of score in Raven’s Progressive Matrices: *b* = 4.52, *t* = 3.44, *p* = .001. This corresponded to a standardised effect size of 0.48, a medium effect. Adjusted *R*^2^ for the model was .21, suggesting that this predictor explained around a fifth of the variance in non-verbal analogy scores. Adding PPVT did not improve either model (for WJVA, *F*(1, 39) = 2.1, *p* = .158; for Raven’s, *F*(1, 39) = 1.7, *p* = .203). Figure 5 shows scatterplots of the relationships between log specific comparisons and the two outcomes. As noted in Aim 2 Method, we also ran analyses using the un-transformed original counts, replicating the analysis in Silvey et al. (2017). The results are similar, and are reported in full in Text S4, Figure S1, and Tables S1 and S2 of the Supplementary Material.

#### *Control Analysis 1*.

Our first control analysis found that log global comparison count was not a significant predictor of score in the Woodcock-Johnson Verbal Analogies: *b* = 0.41, *t* = 0.95, *p* = .349. Log global comparison count was also not a significant predictor of score in Raven’s Progressive Matrices: *b* = 0.09, *t* = 0.05, *p* = .959. PPVT was justified for inclusion in both models.

#### *Control Analysis 2*.

Our second control analysis found that overall number of utterances was not a significant predictor of score in the Woodcock-Johnson Verbal Analogies: *b* < 0.001, *t* = 0.60, *p* = .555. Number of utterances was also not a significant predictor of score in Raven’s Progressive Matrices: *b* < 0.001, *t* = 0.40, *p* = .691. PPVT was justified for inclusion in both models.

Results from all three models for Woodcock-Johnson Verbal Analogies are shown in Table 5, and for Raven’s Progressive Matrices in Table 6.

**Table 5.** Results from models analysing the effect of log specific comparison count (main analysis), log global comparison count (control analysis 1), and total utterance count (control analysis 2) on child score in Woodcock-Johnson Verbal Analogies, controlling for PPVT score at 54 months where this improved the model.

**Model**	**Predictor**	***b***	***β***	***SE***	***t***	***p***
Main	Log specific comparison count	1.29	0.52	0.334	3.86	<.001
Control 1	Log global comparison count	0.41	0.14	0.431	0.95	.349
	PPVT	0.04	0.39	0.014	2.61	.013
Control 2	Total utterance count	0.00009	0.09	0.0002	0.60	.555
	PPVT	0.04	0.42	0.013	2.89	.006

**Table 6.** Results from models analysing the effect of log specific comparison count (main analysis), log global comparison count (control analysis 1), and total utterance count (control analysis 2) on child score in Raven’s Progressive Matrices, controlling for PPVT score at 54 months where this improved the model.

**Model**	**Predictor**	***b***	***β***	***SE***	***t***	***p***
Main	Log specific comparison count	4.52	0.48	1.316	3.44	.001
Control 1	Log global comparison count	0.09	0.01	1.698	0.05	.959
	PPVT	0.14	0.40	0.054	2.60	.013
Control 2	Total utterance count	−0.0002	−0.06	0.0006	−0.40	.691
	PPVT	0.15	0.42	0.052	2.81	.008

#### *Children’s Comparisons and Socio-economic Status*.

Previous work has consistently found a relationship between family SES and children’s language development (Clegg & Ginsborg, 2006). One alternative explanation for the relationship we find between children’s early comparisons and their later analogical reasoning skill is that high levels of both are simply associated with high SES.

To investigate this possibility, we first calculated a composite *z*-score of SES from household income and parents’ years of education. As expected, we found a strong relationship between this measure of SES and log specific comparisons produced by children during the observational sessions: *b* = 0.39, *t* = 3.16, *p* = .003. We also found a strong relationship between SES and children’s scores in our two analogical reasoning tests, the Woodcock-Johnson Verbal Analogies (*b* = 0.98, *t* = 3.28, *p* = .002) and Raven’s Progressive Matrices (*b* = 3.12, *t* = 2.62, *p* = .012). We then ran models that included both our key predictor (log child specific comparison count) and, as a control, the SES *z*-score, predicting children’s scores in each of the analogical reasoning tests. The results are shown in Table 7. Controlling for socio-economic status, children’s specific comparisons were still a significant predictor of outcomes in tests of analogical reasoning. Indeed, once children’s specific comparisons were taken into account, socio-economic status was no longer a significant predictor. This finding suggests that the relationship we found is not simply an epiphenomenon of SES.

**Table 7.** Results from models investigating the effect of log specific comparison count and socio-economic status on children’s scores in analogical reasoning tests (WJVA = Woodcock-Johnson Verbal Analogies; Raven’s = Raven’s Progressive Matrices).

**Outcome**	**Predictor**	***b***	***β***	***SE***	***t***	***p***
WJVA	Log specific comparison count	0.97	0.39	0.361	2.70	.010
	Socio-economic status (*z*-score)	0.60	0.28	0.311	1.94	.059
Raven’s	Log specific comparison count	3.62	0.38	1.455	2.49	.017
	Socio-economic status (*z*-score)	1.73	0.21	1.252	1.38	.176

### Aim 2 Discussion

We replicated the central finding from Silvey et al. (2017) in our larger sample of 42 children. The frequency of specific comparisons in children’s spontaneous speech from 14–58 months predicted children’s scores in verbal and non-verbal tests of analogical reasoning given when children were around 11 years old. Further, this relationship was particular to specific comparisons: neither global comparisons, nor children’s overall number of utterances, showed the same relation with analogical reasoning. The standardised effect of specific comparisons on analogical reasoning scores was around 0.5 for both outcomes, and this predictor explained 20–25% of variance in children’s outcomes.

There are a number of different ways this result could be interpreted. One is that children’s early specific comparisons are a valid index of their developing analogical reasoning skill. Another is that both the prevalence of specific comparisons in early child speech and later scores in analogical reasoning tests are epiphenomena of a third variable, such as children’s overall language competence or their family’s socio-economic status. Our control analyses, and the fact that child vocabulary is not a significant predictor of analogical reasoning skill once specific comparisons are controlled for, argue against the ‘overall language competence’ possibility. Our analyses that control for SES particularly do not support that being the true explanatory variable. Still, we cannot eliminate the possibility that another variable we have not examined explains both our predictor and our outcome. One candidate is the frequency of interaction contexts that may encourage both spontaneous comparisons and the development of relational thinking, such as book reading or playing with building blocks. Since we deliberately allowed interaction context to vary naturally, we cannot easily investigate this possibility in the current dataset, but it should be borne in mind for future work.

If the relation between specific comparisons and analogical reasoning is genuine, there are two possible explanations for this linkage. The first is that children with high latent levels of analogical reasoning skill will naturally tend to produce more specific comparisons in their early spontaneous speech. The second is that the early production of specific comparisons (presumably prompted by some factor in children’s environment) is a causal factor in the development of children’s analogical reasoning skill, in a naturalistic mirror of the results shown by experimental studies. It was this remaining question that led us to Aim 3 of our paper: to investigate the possible influence of parents’ behaviour on children’s production of specific comparisons.

## AIM 3: TO INVESTIGATE PARENTAL INFLUENCE ON CHILDREN’S PRODUCTION OF SPECIFIC COMPARISONS

### Aim 3 Method

#### Data.

Measures of parents’ and children’s production of spontaneous comparisons and other speech were taken from the same longitudinal dataset of 42 children and their caregivers described under Aim 1.

### Variables: Modelling Hypothesis

The modelling hypothesis is that children of parents who more frequently model the production of specific comparisons will go on to produce more specific comparisons themselves. The following variables were calculated to evaluate this hypothesis.

#### *Specific Comparisons Parents Produced During Sessions 1–3*.

The number of specific comparisons each parent produced during the first three observational sessions (child ages 14–22 months) is our predictor of interest for the modelling hypothesis. These sessions precede the earliest comparisons children produced (at 26 months). We can therefore be confident that they represent parental modelling unaffected by children’s own comparisons. As for children, the distribution of this variable is skewed; we therefore log-transform it before running our analysis.

#### *Global Comparisons Parents Produced During Sessions 1–3*.

The number of global comparisons each parent produced during the first three observational sessions is the predictor in our first control analysis for the modelling hypothesis. The distribution of this variable is also skewed; we therefore log-transform it before running our analysis.

#### *Number of Utterances Parents Produced During Sessions 1–3*.

The total number of utterances each parent produced during the first three observational sessions is the predictor in our second control analysis for the modelling hypothesis.

#### *Parent IQ*.

We measured parent IQ when children were in 5th grade, using the FSIQ-4 composite from the Wechsler Abbreviated Scale of Intelligence (WASI-II, Wechsler, 2011). This variable was also used in the analysis for the responsiveness hypothesis below.

#### *Specific Comparisons Children Produced During Sessions 4–12*.

The total number of specific comparisons each child produced during the sessions following the sessions in which parent modelling was measured is our outcome. Note that since no child produced a comparison before session 4, in practice this is identical to the number of specific comparisons produced during sessions 1–12, that is, the predictor variable for the main analysis described under Aim 2. As before, this variable was log-transformed before being entered in the analysis.

#### Variables: Responsiveness Hypothesis

##### *Parent Engagement with Children’s Early Specific Comparisons*.

To create the predictor variable for the responsiveness hypothesis, parents were classified as high-engagement or low-engagement based on their responses to their child’s first specific comparison(s). ‘First specific comparison(s)’ were those the child produced during the first session in which they started producing this type of comparison. For our coding of parent responses, see the full coding scheme in Text S1 of the Supplementary Material. Offering a question, challenge, or elaboration to a child’s specific comparison was classified as a high-engagement response. Simply confirming a child’s specific comparison, or not responding directly at all, was classified as a low-engagement response. We considered parents’ responses to all specific comparisons their child produced during the onset session for this comparison type. Parents were classified as high-engagement if they provided at least one high-engagement response during this session, otherwise they were classified as low-engagement. This variable had one missing value: one child never produced any specific comparisons and so never had the opportunity to elicit any response. Of the 41 parents who could be classified, 16 were classified as high-engagement and 25 as low-engagement.

##### *Specific Comparisons Children Produced in the 2 Sessions Following Onset*.

The outcome for the responsiveness hypothesis was the number of specific comparisons each child produced during the two sessions immediately following the onset session for this comparison type. Again, the distribution of this variable was skewed and so we log-transformed it before running our analyses. This variable had two missing values. As noted above, one child never produced any specific comparisons. Another child produced their first specific comparison in session 11, meaning only one of the two following sessions was available. Missing data for this and the previous variable were imputed via multiple imputation using the mice library in R (van Buuren & Groothuis-Oudshoorn, 2011). Full details are provided in Text S3 of the Supplementary Material.

The predictor and outcome for the responsiveness hypothesis thus constitute a relatively small slice of data (one session for the parents, and two sessions for the children). We chose this procedure in order to maximise the size of our sample: the fact that many children start producing specific comparisons relatively late constrains the number of sessions within which we can measure parent responsiveness and subsequent child behaviour. We acknowledge the limitations of this approach in the Aim 3 Discussion.

Aim 3 is to ask whether children’s production of specific comparisons—our predictor of interest for Aim 2—was influenced by parents’ behaviour. The research questions and analysis plan for Aim 3 are documented in a preregistration on the Open Science Framework (https://osf.io/gaq97). We investigate two ways in which parents could influence their children’s production of specific comparisons: modelling, where parents produce specific comparisons prior to children’s onset of this behaviour, and responsiveness, where parents respond to their children’s earliest specific comparisons in more or less engaged ways. For the modelling hypothesis, we run three analyses: 1) our main analysis, predicting the number of specific comparisons children produced during observational sessions 4–12 from the number of specific comparisons their parents produced during the first three sessions; 2) our first control analysis, predicting the number of specific comparisons children produced during observational sessions 4–12 from the number of *global* comparisons their parents produced during the first three sessions; 3) our second control analysis, predicting the number of specific comparisons children produced during observational sessions 4–12 from the total number of utterances their parents produced during the first three sessions. The first control analysis accounts for the possibility that, if we find a relationship between specific comparisons produced by parents and their children, this is actually driven by parents’ production of comparisons in general. The second control analysis accounts for the possibility that, if we find a relationship between specific comparisons produced by parents and their children, this is actually driven by parents’ overall talkativeness. As in the control analyses for Aim 2, we run these models separately, rather than entering all three predictors into the same model, because we are not trying to test which of these three predictors explains the outcome over and above the others when considered simultaneously. Rather, we are testing distinct hypotheses about whether a more general construct, such as overall comparisons or overall talkativeness, predicts our outcome as well as our theoretically motivated predictor, specific comparisons. For the responsiveness hypothesis, we run one analysis, predicting the number of specific comparisons a child produced during the two sessions after onset of this behaviour from whether their parent responded to their earliest specific comparisons in a high- or low-engagement way. Parent IQ was included as a covariate in each of our models to control for genetic and environmental influences on children’s tendency to produce specific comparisons.

All analysis code and de-identified data are available at https://github.com/silveycat/comparisons.

### Aim 3 Results

All models reported below control for parent IQ, as planned in our preregistration. Full results are shown in Tables 8 and 9.

**Table 8.** Results from models analysing the effect of log specific parent comparison count in sessions 1–3 (main analysis), log global parent comparison count in sessions 1–3 (control analysis 1), and total parent utterance count in sessions 1–3 (control analysis 2) on log child specific comparison count in sessions 4–12, controlling for parent IQ.

**Model**	**Predictor**	***b***	***β***	***SE***	***t***	***p***
Main	Log specific comparison counts sessions 1–3 (parent)	0.09	0.08	0.160	0.54	.592
	Parent IQ	0.02	0.29	0.010	1.84	.074
Control 1	Log global comparison count sessions 1–3 (parent)	0.30	0.34	0.135	2.25	.030
	Parent IQ	0.01	0.19	0.010	1.23	.227
Control 2	Total utterance count sessions 1–3 (parent)	0.0001	0.23	0.00009	1.45	.155
	Parent IQ	0.015	0.24	0.010	1.52	.138

**Table 9.** Results from model analysing the effect of parent responsiveness (high-engagement vs. low-engagement) to children’s earliest specific comparisons on log child specific comparison count in the following two sessions, controlling for parent IQ.

**Predictor**	***b***	***SE***	***t***	***p***
Parent responsiveness	0.086	0.273	0.31	.755
Parent IQ	0.004	0.012	0.36	.721

#### Modelling Hypothesis.

Our main analysis found that parent log specific comparison count during the first three sessions was not a significant predictor of child log specific comparison count during the remaining sessions: *b* = 0.09, *t* = 0.54, *p* = .592. Parent IQ, included in the model as a control, was a marginal predictor of the outcome: *b* = 0.02, *t* = 1.84, *p* = .074.

Our first control analysis found, unexpectedly, that parent log global comparison count during the first three sessions was a significant predictor of child log specific comparison count during the remaining sessions: *b* = 0.30, *t* = 2.25, *p* = .030. Parent IQ, included in the model as a control, was not a significant predictor: *b* = 0.01, *t* = 1.23, *p* = .227. Comparing the model with parent log global comparison count and parent IQ to a simpler model with parent IQ only, we find an effect size of *f*^2^ = 0.11. This exceeds the threshold of 0.07, which we set in our preregistration as the smallest effect size we would consider meaningful. We consider the implications of this unexpected result in the Aim 3 Discussion.

Our second control analysis found that total parent utterance count during the first three sessions was not a significant predictor of child log specific comparison count during the remaining sessions: *b* = 0.0001, *t* = 1.45, *p* = .155. Parent IQ was not a significant predictor in this model: *b* = 0.015, *t* = 1.52, *p* = .138.

**Figure 6.**
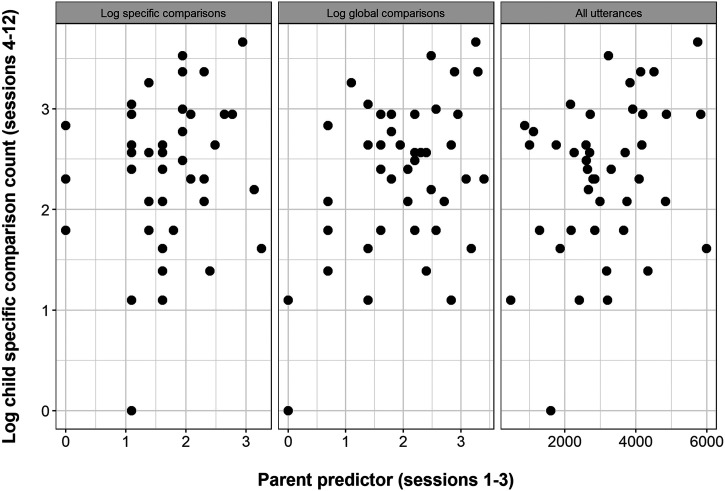
Scatterplots showing the relationship between the three predictor variables from our main and control analyses and our outcome, log child specific comparison count in sessions 4–12.

Results from all models are reported in Table 8. Figure 6 shows scatterplots of the predictor variables from the three models against the outcome variable.

#### *Responsiveness Hypothesis*.

Our dataset for investigating the effect of parent responsiveness includes missing values (see Aim 3 Method for details). We therefore report analyses run on 5 imputed datasets, with the results pooled according to Rubin’s rules (Rubin, 1987). Details of the imputation are reported in Text S3 of the Supplementary Material.

**Figure 7.**
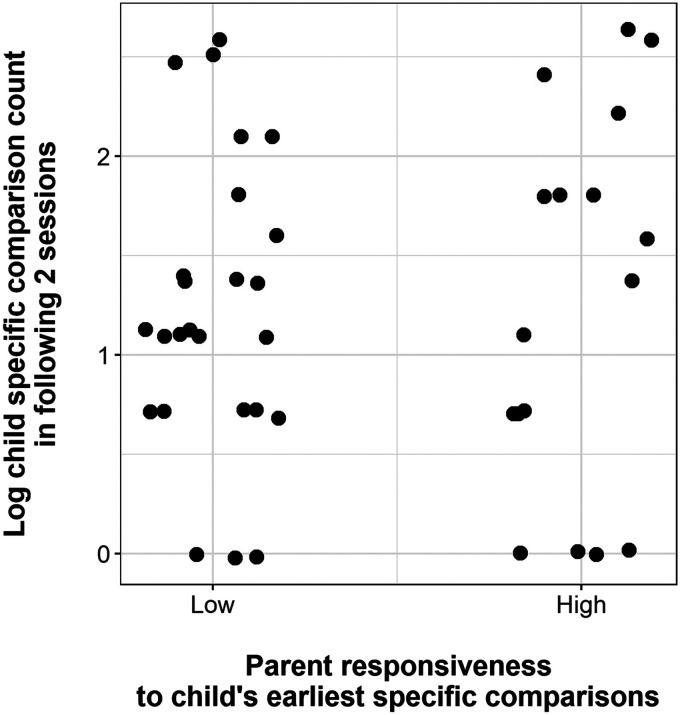
Scatterplot showing the relationship between parent responsiveness (low vs. high engagement) to children’s earliest specific comparisons and log child specific comparison count in the immediately following two sessions. Points are jittered to avoid overplotting.

Parents’ responsiveness to their children’s earliest specific comparisons did not significantly predict child log specific comparison count in the following two sessions: *b* = 0.086, *t* = 0.31, *p* = .755. Parent IQ, included as a control, was also not a significant predictor in this model: *b* = 0.004, *t* = 0.36, *p* = .721. Figure 7 shows a scatterplot of the distribution of children’s log specific comparison count in the two sessions after onset, categorised by whether their parents’ responses during the onset session were categorised as high- or low-engagement.

#### Exploratory Analyses.

Below, we report the results of a number of exploratory analyses of the data. Further exploratory analyses examining the relationship between spatial comparisons and child gender, and how children’s and parents’ comparisons change over time, are reported in Text S5 and Figures S2–S7 of the Supplementary Material.

##### *Parents’ Global Comparisons and Children’s Specific Comparisons: Alternative Explanations*.

Our first exploratory analysis investigated one possible explanation for the unexpected finding from our confirmatory analysis. Although parents’ specific comparisons in the first three sessions did not significantly predict children’s specific comparisons in later sessions, parents’ global comparisons did. Global comparisons were overall more frequent than specific comparisons in parent input during these early sessions (Figure 4), which means that the correlation between log global comparison count and log overall comparison count in these sessions is very high (*r* = .93). One possible interpretation of this finding, then, is that *overall* parent comparison input is what actually predicts children’s later production of specific comparisons.

We tested this hypothesis by running an analysis where we used log overall parent comparison count during the first three sessions (i.e., the log-transformed total of specific comparisons plus global comparisons) as our predictor, again controlling for parent IQ. Despite the high correlation between global and overall comparisons, log overall parent comparison count was not a significant predictor of log child specific comparison count during the remaining sessions: *b* = 0.227, *t* = 1.51, *p* = .139. This suggests that, if the effect we find is real, it is driven by global comparisons in particular, rather than by comparison input overall. Potential reasons for this are proposed in the Aim 3 Discussion.

##### *Parents’ Comparisons in Relation to IQ*.

We controlled for parent IQ in our main analyses; now we directly examine whether IQ relates to our key measures of parent comparison: modelling (production of specific comparisons during the first 3 sessions) and responsiveness (high- vs. low-engagement response to children’s earliest specific comparisons). Parent IQ was not a significant predictor of log specific comparisons during the first 3 sessions: *b* = 0.016, *t* = 1.69, *p* = .098. For parent responsiveness to early child comparisons, IQ did not significantly differ for low- versus high-engagement parents: *b* = 3.95, *t* = 1.02, *p* = .317.

We also examined the relationship between parent IQ and parents’ global comparisons during the first 3 sessions, since we had found an unexpected relationship between this control variable and children’s specific comparisons. Parent IQ significantly predicted log global comparisons produced during the first 3 sessions: *b* = 0.025, *t* = 2.40, *p* = .021. Adjusted *R*^2^ for the model was .10: while IQ was associated with early production of global comparisons, it only accounted for a small amount of variance. We consider this finding in the Aim 3 Discussion.

##### *Progressive Alignment and Children’s Specific Comparisons*.

The theory of progressive alignment (Gentner et al., 2011; Kotovsky & Gentner, 1996) states that children benefit from being exposed to high-similarity pairs before low-similarity pairs. The reasoning is that children can easily align high-similarity pairs, allowing them to extract a common partially abstracted relational schema, which they can then recognise even in low-similarity pairs. In relation to the current study, this theory suggests that it should be more helpful for children’s development if their parents produce within-category comparisons before producing between-category comparisons.

In our description of parents’ comparisons under Aim 1b, we found that parents were evenly split: half produced within-category comparisons before between-category comparisons (suggesting progressive alignment), while half did not make an ordering distinction. Did the children of parents who performed progressive alignment go on to produce more specific comparisons? We classified children according to whether their parents performed progressive alignment and used this binary classification to predict the log number of specific comparisons children produced. We found no clear effect of progressive alignment on children’s specific comparisons: *b* = −0.02, *t* = −0.09, *p* = .932.

### Aim 3 Discussion

Our preregistered analyses tested two hypotheses: the modelling hypothesis and the responsiveness hypothesis. We found support for neither hypothesis. The prevalence of specific comparisons in parents’ input to children from 14–22 months did not significantly predict children’s subsequent production of specific comparisons. Parents’ responsiveness to their children’s earliest specific comparisons (i.e., whether parents were classified as high-engagement or low-engagement) also did not predict the prevalence of specific comparisons in children’s speech in the immediately following sessions. In both cases, the predictors were neither significant on their own, nor when controlling for parent IQ. As we did for Aim 2, we ran planned control analyses to account for the possibility that comparisons in general, or overall parent talkativeness, underlay any relation we might have found. Importantly, overall parent talkativeness between child ages 14 and 22 months did not significantly predict children’s production of specific comparisons in the following sessions.

However, we found an unexpected and significant relationship between the prevalence of *global* comparisons in parents’ input to children from 14–22 months and children’s subsequent production of specific comparisons. This relationship was significant without controlling for parent IQ; when parent IQ was controlled for, parent production of global comparisons remained a significant predictor, and parent IQ was no longer a significant predictor. Followup analyses suggested that this effect was not driven by overall comparison input (i.e., specific + global), but by global comparisons in particular. The variance explained by global comparisons over and above IQ corresponded to an *f*^2^ of 0.11, a small-to-medium effect (Cohen, 1988). Additionally, although we did not find a significant relationship between parent IQ and either our modelling or our responsiveness predictor, we did find a significant relationship between parent IQ and parent production of global comparisons from child age 14–22 months. As a caveat, it is important to note that a) the relationship between parents’ global comparisons and children’s specific comparisons holds even when controlling for parent IQ, and b) the proportion of variance in global comparisons explained by parent IQ is small, around 10%. Thus, it does not appear that the relationship between parents’ global comparisons and children’s specific comparisons is fully explained by parent IQ.

Although this control analysis was planned as part of our preregistration, we did not expect this finding. Furthermore, it is one significant result out of many analyses, raising the possibility of a Type I error. For these reasons, it should be interpreted with a great deal of caution. However, one possible interpretation is that parent comparison input does influence children’s production of specific comparisons, but not in the way predicted by the modelling hypothesis. Specific comparisons may be too sophisticated or abstract to be useful input for children at the age of 14–22 months; at this age, global comparisons may function to invite children into the comparison process in a more accessible way, laying the groundwork for them to develop more sophisticated forms of comparison in their own speech. If this interpretation holds, it potentially reinforces Özçalışkan and colleagues’ (2009) conclusion that learning the word ‘like’ is an important precursor to producing specific comparisons: Hearing children did not start producing specific comparisons until they began producing ‘like’; and deaf homesigners, who did not have a gesture for ‘like,’ produced only global comparisons and not specific comparisons. ‘Like’ is implicated in our study in the role it plays in parents’ language input: 69% of parents’ global comparisons overall used the word ‘like’ (rising to 78% during the crucial first three sessions), whereas the prevalence of this word was much lower in parents’ specific comparisons, appearing in only 14% of these utterances (17% during the first three sessions). For individual parents during the first three sessions, the number of global comparisons they produced and the number of comparisons using ‘like’ they produced had a correlation of *r* = .97. Thus, it may be parents’ use of the word ‘like’, rather than their use of global comparisons, which fosters their children’s development of specific comparison and later analogical reasoning. However, this interpretation is necessarily speculative, given the limited nature of our data, and should be followed up by experimental work in large samples.

## GENERAL DISCUSSION

This paper makes three main contributions. Firstly, we describe the characteristics of the comparisons parents and children produce in their spontaneous speech in the home when children are between 14 and 58 months old, uncovering trends in the topics, features, and ordering of these comparisons to shed new light on the development of this precursor of analogical reasoning. Secondly, we demonstrate a robust relationship between the prevalence of a particular type of comparison—specific comparisons, which point out a feature of similarity or difference—in children’s early speech and children’s later analogical reasoning skill, as measured by standardised tests in 6th grade. Thirdly, we offer a speculative path by which parents’ early production of more accessible global comparisons—or perhaps the comparison word ‘like’—may bootstrap children’s ability to later produce specific comparisons, suggesting a link between parental input and children’s analogical reasoning skills.

What should we conclude from the null results of our preregistered analyses testing the effect of parent modelling and responsiveness on children’s production of specific comparisons? Parent modelling and responsiveness in general have robust effects on child language, as two recent meta-analyses have reinforced (Madigan et al., 2019; Anderson et al., 2021). Why then do we not see these effects for specific comparisons? One possibility is that children’s comparison development is not affected by parent modelling or responsiveness in the same way as language in general. But it could simply be that the relative rarity of comparison, along with the difficulty of operationalising it consistently (as shown by the number of disagreements in our coding), makes it difficult to find robust associations between parent and child behaviour in naturalistic observational samples. Previous studies that found effects of parent modelling and responsiveness have used ubiquitous and easily measured variables, such as number of unique words used during a session, or children’s scores on standardised tests. Our null results should therefore be interpreted with caution. Experimental studies will be crucial for conducting more robust tests of these hypotheses in future.

There are several further caveats to the results we report. The first is that we have a small sample of only 42 parent-child pairs. The small sample constitutes a further reason for caution in interpreting null results (since we have weak power to detect small effects), and also means that any significant results we find should ideally be replicated in a larger sample. The second concerns the generalisability of the results beyond our sample. While the original sample of 64 children was selected to be representative of the demographics of the greater Chicago area at the time of data collection, by focusing on a subset of the participants for whom we have complete data, we have likely introduced selection bias, since lower-SES families were more likely to drop out of the study before the observational sessions were complete. The third is that, while the long time-gap between the early observational sessions and the 6th grade analogical reasoning outcomes makes the strong relation we find more surprising, it also means we are unable to rule out the influence of factors that occur during the period in between. The fourth is that we do not control for cognitive factors that are known to influence the development of analogical reasoning and could also influence children’s ability to produce specific comparisons in interactional contexts, most notably working memory (Simms et al., 2018).

Two more specific caveats apply to our test of the responsiveness hypothesis, and our exploratory investigation into the possible effect of parents performing progressive alignment. For the responsiveness hypothesis, although we tried to find a predictor and outcome that constituted the most valid and robust test of this hypothesis in our data, there are many other potential ways the hypothesis could have been formalised. Rather than testing them all and selectively reporting the one that worked, we chose to preregister one and report the results; however, this should not be taken as a conclusive rejection of the hypothesis, particularly as the method we chose only analyses a small slice of the data. A similar concern applies to our test of whether the children of parents who perform progressive alignment go on to produce more specific comparisons. Our measure of progressive alignment, based on classifying parents according to whether they produced within-category comparisons before between-category comparisons, is likely to be extremely noisy. With each session only capturing 90 minutes of interaction and 4 months between sessions, a significant number of parents may have been miscategorised on the basis of sparse data. This concern also applies to the cases where we find null effects of ordering of different comparison types. It could be that, in these cases, different comparison types appear simultaneously simply because our observation period can only capture a subset of a parent’s or child’s behaviour at a particular time.

Despite its limitations, this study provides new insights into the details of children’s comparison language and inputs, and we hope it acts as a catalyst for future research. In particular, we see rich potential for experimental work that uses the features of spontaneous comparison to test follow-up hypotheses in a way that is firmly tied to children’s real language experience.

## CONCLUSION

Experimental studies have demonstrated that prompting children to make comparisons has a beneficial effect on their analogical reasoning skills. We built on this work by investigating the spontaneous comparisons that children and their parents make in the course of naturalistic interactions in the home. The results increase our knowledge of how comparison develops in real-world contexts, provide evidence that children’s early specific comparisons are a reliable index of later analogical reasoning skill, and suggest an avenue by which parents may be able to encourage their children’s production of specific comparisons by offering more accessible global comparisons using the word ‘like’ as a model before children begin producing comparisons themselves. By taking research on comparisons from the lab to the home, we ground previous work in the real world and open up avenues for future experiments testing our findings.

## ACKNOWLEDGMENTS

We thank Kristi Schonwald and Naureen Hemani-Lopez for administrative support, Anjali Murthy for data coding, the research assistants on the Language Development Project for data collection/transcription, and the families for their participation. We also thank the attendees of the Analogy 2017 conference for helpful discussions.

## AUTHOR CONTRIBUTIONS

Contributed to conception and design: CS, DG, LR, SG-M. Led the acquisition of data: SG-M. Led the analysis of data: CS. Contributed to interpretation of data: CS, DG, LR, SG-M. Drafted and/or revised the article: CS, DG, LR, SG-M. Approved the submitted version for publication: CS, DG, LR, SG-M.

## FUNDING INFORMATION

This research was supported by NICHD, P01-HD40605 (PI: SG-M) and The Successful Pathways from School to Work Initiative, funded by the Hymen Milgrom Supporting Organization. DG’s participation was supported in part by the Spatial Intelligence and Learning Center (SILC) at Northwestern University. CS was supported by a British Academy Postdoctoral Fellowship.

## DATA AVAILABILITY STATEMENT

The de-identified data that support the findings reported under Aim 2 and Aim 3 of this study are openly available at https://github.com/silveycat/comparisons. Data that support the findings reported under Aim 1 cannot be made openly available, since these data by their nature could not be de-identified and participants did not consent for their data to be publicly shared.

## Notes

^1^ Özçalışkan et al. focused on the characteristics of the objects being compared (i.e., whether the objects shared a single feature or multiple features) to determine whether a comparison was specific or global. Since our dataset includes older, more linguistically advanced children, we focus instead on the language used in the comparisons (i.e., whether a feature of similarity or difference such as ‘same colour’ is verbally specified).^2^ To ensure data are de-identified, all utterance examples are invented for this paper. However, they are closely based on real utterances from the transcripts.^3^ Comparisons using the word ‘favorite’ were not coded, since it was not clear that children understood its meaning as comparative either in parents’ speech or when producing it themselves.^4^ One child did not produce any comparisons until the final session, and so onset by this criterion could not be determined for this child.^5^ For all ordering analyses, we exclude participants who produced both comparison types during the same session and contrast only the two possible orderings (e.g., specific-first vs. global-first).^6^ Özçalışkan et al. (2009) used a criterion for global and specific comparison based on the number of features the objects shared, whether or not the features were expressed in language. The authors used this criterion to facilitate comparison to deaf homesigners (profoundly deaf children, unable to learn spoken language and not exposed to sign language, who used gesture to communicate); the homesigners used pointing gestures to make their comparisons.^7^ We also run untransformed analyses to replicate the original result. These are reported in Text S4, Figure S1, and Tables S1 and S2 of the Supplementary Material.
