# Supplementary material for: Children’s Early Spontaneous Comparisons Predict Later Analogical Reasoning Skills: An Investigation of Parental Influence
Source: Open Mind (Camb). 2023 Jul 28;7:483–509. doi: 10.1162/opmi_a_00093 (PMC10449400; doi:10.1162/opmi_a_00093)
Supplement: Supplementary file 1 [file opmi-07-483-s001.docx]

**Supplementary Material**

**Text S1.** Full coding scheme for parent and child comparisons.

Coding was done from 2 sources:

- the transcript context for each comparison, containing the 5 utterances before and after the utterance containing the comparison

- the video for the corresponding observation session

The basis for all coding was identifying the source and the target of the comparison: the two objects or events being compared. Where possible, these were coded from the utterance and its context in the transcript. Where the identity of the source and target were not clear from the transcript, we consulted the video for the session.

Where an utterance contained more than one comparison (e.g. ‘This is prettier because it’s shinier’), each comparison was coded separately.

**Categories coded:**

**Session**: Which of the 12 home visits the utterance is from. As noted in the paper, the 12 visits corresponded to child ages 14, 18, 22, 26, 30, 34, 38, 42, 46, 50, and 54 months.

**Speaker**: Parent = parent produced the utterance containing the comparison; Child = child produced the utterance containing the comparison.

**Word**: the key word that made the utterance a comparison. The most common were:

like

comparative/superlative adjectives, e.g., easier, best. This included ‘more’ in comparative contexts such as ‘that’s more important’. ‘Favorite’ was counted as a comparative/superlative, but comparisons that used ‘favorite’ only were excluded from further coding – see Exclusions section below.

too (in sense of ‘too hot’ etc., or in sense of ‘that one’s blue too’)

both

either

match

same

different

else

another

instead

similar

alike

as (e.g., ‘as big as an elephant’)

Sometimes, there was no specific word that made the utterance a comparison - e.g., ‘ice cream tastes good but cars don’t taste good’ draws a comparison without using a specific comparison word. Here, Word would be coded as NA.

If two comparison words were used to make the same comparison, we coded the first word used. e.g., ‘it smells the same like that one’ = same; ‘I’m happy too like Pooh’ = ‘too’.

**Word Category**: a more broad categorization of the Word column. The categories were:

like (for like or alike)

compsup (for any comparative or superlative adjective, including more/most)

too (for either sense of too)

samediff (for same or different)

match (for match)

other (for any other word, including NA = no specific comparison word used)

**Source**: The source of the comparison, i.e. the thing being compared to.

**Target**: The target of the comparison, i.e., the thing being compared.

Some clarifying examples of which is which:

‘twinkle twinkle little star…like a diamond in the sky’ Source = diamond, Target = star

‘can you run around like a doggy?’ Source = dog running, Target = child running

Note: in some comparative questions, part or all of either Source or Target could be left open. In these cases, we used a question mark to denote the open element. Examples:

‘who does he look like?’ Source: ? Target: He

‘who’s the tallest [in the family]?’ Source: people in family, Target: ? person in family

‘what does salt taste like?’ Source: ? Target: salt

**Provider**: Whether the parent or the child explicitly made the comparison. This was not always redundant with Speaker. The original higher-order thinking coding scheme that the comparisons coding was based on gave children credit for responding to comparative questions – e.g., responding to ‘Does that look like her?’ with ‘yes’. In this case, the original provider of that comparison was actually the parent, and the child did not actually produce a comparison themselves. For our coding scheme, child utterances where Provider = Parent were not included as child comparisons. However, if the child did use a comparison word in their own utterance (e.g., if the child had responded, ‘yes, that looks like her’), then Provider would be coded as Child, even if the parent introduced this comparison first.

**Similarity/Difference**: Whether the utterance expressed a similarity between two objects/events or a difference between two objects/events.

Examples:

‘dad look I can sit like a dog’ – Similarity

‘I think it is too’ - Similarity (what the parent thinks and what the child thinks are implied to be similar)

‘I’m too big for that’ – Difference (a contrast is being drawn between the child’s size and the size that would be appropriate)

‘I want bigger toys’ - Difference (a contrast is being drawn between the size of the actual toys and the size of the toys the child wants - comparatives/superlatives were always coded as Difference)

**Object/Event**: Whether the source and target being compared were objects or events. Event was used in a broad sense to mean any comparison that went beyond comparing a single object to a single object. For example, comparisons of relations between groups of objects (like amounts or spatial relations), and comparisons of relations between people and objects (e.g., someone preferring something to something else), were both coded as Event.

Examples of some edge cases and how they were categorized:

‘Which one do you like the best?’ – this was coded as Event, since the comparison is not directly between the objects but between the addressee’s preference for one object and another.

Comparisons involving words, letters, and numbers (except amounts) were coded as Object.

‘Looks like we’re in a bubble’ – this was coded as Event, since even though it described a static state, it was about a relation between objects.

Comparisons involving wearing clothes were coded as Event.

Comparisons involving possessing something external to a person were coded as Event; however, comparisons involving having a body part/a beard/something intrinsic to a person were coded as Object.

Comparisons involving smell or taste were coded as Object, since the transcripts suggested that in parent-child conversations these were most often conceptualized as intrinsic features of objects.

Comparisons involving sounds were coded as Event.

Comparisons involving games, shows or movies were coded as Event.

Comparisons involving amounts were coded as Event.

Comparisons where an object was compared to an event (e.g., a parent looking at a child’s drawing and saying ‘that looks like a party’) were coded as Event.

Comparisons involving a hypothetical or real change of state of an object (e.g., ‘you’re getting bigger’, ‘we’ll make it better’) were coded as Object, since they were comparing current or future states of the same object.

Comparisons involving age were coded as Object if age was mentioned in isolation (e.g., ‘you’re older than him’). However, if used in a context that implied an event (e.g., ‘you’re too old for that movie’), then these comparisons were coded as Event.

‘too X to Y’/ ‘X enough to Y’ was always coded as Event, since it implies being too X/ X enough for a particular action or relation: e.g., ‘is the chair too big for you?’ ‘too steep for your bike’, ‘strong enough to hold us both’, ‘you’re not small enough for the seat’.

**Within/Between Category**: Whether the objects involved in the comparison were from the same superordinate category or different superordinate categories.

For Object comparisons, this related to the two objects being compared. For Event comparisons, this related to all the objects taking part in the event. If the objects in corresponding roles in the Source and Target of the comparison were from the same superordinate category, the comparison was coded as Within Category.

The superordinate categories used (based on Özçalışkan et al., 2009) were:

people, animals, body parts, vehicles, clothing, furniture, appliances, kitchen utensils, tools, musical instruments, food, plants, activity toys, places, decorations/crafts, words/letters, shapes

In cases where it was difficult to confidently assign all objects in a comparison to these categories, we took the following steps:

- If the Source or Target object(s) had been assigned to a category, and the other objects clearly did not belong to the same category, the comparison was coded as Between Category.

- If neither the Source nor the Target object(s) could be assigned to a superordinate category, but were in the same basic-level category (e.g., a box and another box), the comparison was coded as Within Category.

- If neither the Source nor the Target object(s) could be assigned to a superordinate category, and they were not in the same basic-level category, the comparison was coded as NA and excluded from the Within/Between Category analyses.

Some examples:

‘see if it works any better.’ [talking about a pen lid working to open one paint bottle, vs. how well it worked on a previous bottle]. Here, both events being compared concern a pen lid opening a paint bottle, so this was coded as Within Category.

‘so those two words have the same sound?’ Sound was coded as Event; both source and target refer to words, which are in the same superordinate category, so again this was coded as Within Category.

‘I like daisies.’ ‘I like ladybugs too.’ In this comparison, the objects in the “liker” role across Source and Target were in the same category (i.e., people), but the objects in the “liked” role were not (daisies are plants and ladybugs are animals). Therefore this comparison was coded as Between Category.

Constructions like ‘you’re too small for this seat’ were coded as Within Category, since the utterance is not directly comparing the child to the seat, but to a child of appropriate size to fit in the seat.

Future or hypothetical states of the same object (e.g., ‘you’re getting bigger’) were also coded as Within Category.

Toys, drawings, etc. representing real-world objects were coded as belonging to the same superordinate category as the object they represented: e.g., dolls were coded as people, and toy trucks as vehicles.

Geographical features like sky, mountain etc. were coded as places.

For open comparison questions, e.g., ‘what does this one look like?’, the empty Source was considered to have no superordinate category, and so the comparison was coded as Between Category.

Comparisons where the source or target was a gesture were always coded as Between Category, since the gesture represented an object or action abstractly, rather than directly involving any objects.

**Global/Specific**: is the comparison global (expressing an overall similarity or difference between objects/events), or is a specific feature of comparison specified?

For objects, common features specified include color, shape, size, etc.

For events, common features specified include difficulty, goodness, preference, etc.

Note: the specific feature doesn’t have to be mentioned in the current utterance, if it’s clear from the previous discourse. e.g., Parent: ‘it’s bright.’ Child: ‘like the moon.’ Even though the child’s comparison doesn’t express brightness, it’s clear from the previous utterance that this feature is the basis of the child’s comparison.

**Feature specified**: For specific comparisons, which feature was specified. Features were coded into 6 categories:

Evaluative - making a value judgement of some kind. e.g., saying things are better, cooler, safer, nicer, easier, more difficult, prettier, worse, etc.

Physiological - bodily/mental states that aren’t directly perceptible. e.g., strength, tiredness, meanness, sadness, laziness, health, soreness.

Preference – someone liking something more/less than something else

Sensory (e.g., color, taste, smell, temperature, weight, texture)

Spatial (e.g., size, shape, height, distance, position, speed, orientation)

Other (e.g., age, spelling of words, amount, category, anything else that doesn’t fall into the categories above)

Note: if the comparison is about who likes something better, or which thing someone likes better, even though the Word is ‘better’, the Feature is Preference, not Goodness. We only use Goodness when the comparison is about the judged goodness of something.

**FeatureType**: For specific comparisons, we further coded the feature specified into two categories:

P: Perceptual feature. Anything that can be directly apprehended via the senses: e.g., color, size, speed, taste, shape.

NP: Non-perceptual feature. Anything that can’t be directly apprehended via the senses: e.g., difficulty, spelling, preference, emotion, goodness, age, tiredness etc.

**Exclusion**: We excluded utterances that fell into the following categories:

Source and target not codable: Sometimes, source and target were not clearly identifiable even after consulting the video. In these cases, we excluded the utterance from our analysis.

Not a comparison: These were cases when we determined on closer inspection that the utterance tagged as a comparison was not truly comparative. Examples are: idiomatic uses of phrases such as ‘seems like’, ‘looks like’, and ‘different story’; ‘like’ used as a discourse marker (e.g., ‘it was like two hundred feet long’); ‘like’ used to introduce an example, in the sense of ‘such as’ (e.g., ‘It’s just for elephants like Charlie’); ‘same’ used to mark identity, not comparison (e.g., ‘that’s the same hat you wore yesterday’); ‘different’ used to mean ‘another/an alternative’ rather than drawing a contrast, e.g., ‘try a different one’.

‘Favorite’: Comparisons where the only comparison word was ‘favorite’ were excluded, since it was not clear that children who used ‘favorite’ understood the word in a truly comparative sense. For consistency, we also excluded parents’ comparisons where the only comparison word used was ‘favorite’.

Repetition: If the same comparison (i.e., a comparison involving the same source and target) was repeated by the same speaker within five rows of the transcript, we excluded this comparison from our analysis. If the source and target were reversed, i.e. the original source was now the target and vice versa, the comparison was not excluded but was coded as normal.

Imitation: If a comparison constituted a direct imitation of the interlocutor’s previous utterance, and the source and target remained unchanged, we excluded this comparison from our analysis.

**For Child comparisons only:**

**Parent response**: For each comparison a child produced, we coded how the parent responded by checking the following rows of the transcript. Most often, there was one clear response; where multiple utterances could feasibly be responding to the child’s comparison, we coded the latest response. Responses were coded into the following categories:

Confirmation: parent agrees with/repeats child comparison. e.g., Child: ‘I’m taller than everybody!’ Parent: ‘You are.’ / Child: ‘it’s black like my hair.’ Parent: ‘it is black like your hair.’ Very simple agreements/affirmations such as ‘yes’, ‘ok’, ‘go ahead’ also count, as do implicit confirmations like ‘I know’. For the purposes of our parental responsiveness hypothesis, this was coded as a low-engagement response.

None: Parent either doesn’t respond to the child’s comparison, or their response is noncommittal/unrelated to the child comparison’s. e.g., Child: ‘there’s jelly inside there too.’ Parent: ‘oh.’ / Child: ‘we’re both tall.’ Parent: ‘oh, I think your sister left some Legos under there.’ For the purposes of our parental responsiveness hypothesis, this was coded as a low-engagement response.

Question: Parent questions child comparison. e.g., Child: ‘like — our dog.’ Parent: ‘it looks like our dog?’ / Child: ‘it’s like a car.’ Parent: ‘a car?’ This is for questions that don’t request any further information from the child; questions that do request further information would be coded as Elaboration. For the purposes of our parental responsiveness hypothesis, this was coded as a high-engagement response.

Challenge: Parent rejects child comparison or provides alternative. e.g., Child: ‘it’s nicer than mine.’ Parent: ‘I don’t think so.’ / Child: ‘Let’s see if they’re bigger.’ Parent: ‘no, about the same.’ For the purposes of our parental responsiveness hypothesis, this was coded as a high-engagement response.

Elaboration: Parent adds details/requests details from child. e.g., Child: ‘oh yeah the same colour as my shoes.’ Parent: ‘oh and what colour is that?’ For the purposes of our parental responsiveness hypothesis, this was coded as a high-engagement response.

**Text S2**. Coding procedure, reliability, and examples of disagreements.

Coding was done by two coders. One coder coded the comparisons produced by 24 children and 15 parents. The other coder coded the comparisons produced by 18 children and 27 parents. Both coders were aware that specific comparisons were the main variable of interest. However, the Aim 2 outcome measures for each child (i.e. scores in analogical reasoning tests) were not available to coders at the time of coding. For the purposes of the Aim 3 analyses, both coders were aware of the general intention to relate parents’ comparisons to children’s comparisons, but the specific way this would be operationalised in terms of modelling and responsiveness had not yet been decided at the time that coding took place.

To assess whether the coding scheme was being applied reliably, both coders independently coded all comparisons produced by 5 randomly selected parent-child pairs. Where the coders disagreed, the judgement of the more senior coder was accepted, since this coder had more experience with the dataset and could often provide clarifying context (for example, that the referent of an unclear ‘you’ was most likely the experimenter).

A substantial proportion of all disagreements (37 of 91 for children, and 107 of 398 for parents) regarded whether or not a particular comparison should be coded at all, or should be excluded. For children, most frequently these disagreements arose from the more senior coder being more conservative about which utterances were genuine comparisons, and from the more junior coder being unclear about some exclusion criteria (e.g., that if two adjacent comparisons had the same source and target, the second would be excluded as repetition even if it were phrased slightly differently). For parents, most frequently these disagreements arose from the difficulty of applying exclusion criteria consistently to parents’ more complex speech. For example, the coding scheme specified that when the word ‘like’ was used as a particle rather than as a genuine comparison word (‘and you could make, like, a Lego house’), the comparison should be excluded, but this was sometimes difficult to determine. To assess whether these disagreements asymmetrically affected the number of specific comparisons that were counted, we looked at the comparisons that were coded as specific by one coder but excluded by the other. For children, there were 15 of these in total; 6 were resolved in favour of being coded, and 8 were not. For parents, there were 31 of these in total; 20 were resolved in favour of being coded, and 11 were not. While it is hard to draw conclusions from such small numbers, this tentatively suggests that if there was an effect of coding unreliability on the number of specific comparisons, it may consist of an undercounting of the specific comparisons produced by the parents coded by the more junior coder. The presence of this noise in our coding is one of the reasons we urge caution in the interpretation of our results, and the necessity of following up the hypotheses we generate with appropriate experimental work.

The remaining disagreements were on specific coding categories. Below, we characterise the most frequent sources of disagreement for each category and give examples.

*Word category.* The most frequent source of disagreement for this category was the words ‘more’ and ‘most’ not being categorised as comparative/superlative by the more junior coder.

*Similarity vs. difference.* The most frequent source of disagreement for this category was confusion over whether a negative word in the utterance applied to the comparison or not. An example of the former is ‘this part does not seem like that’. The more junior coder coded this as a similarity comparison, whereas the senior coder coded it as a difference comparison on the argument that the overall effect of the utterance is to draw a contrast. An example of the latter is ‘just like I can’t jump over you’. The junior coder coded this as a difference comparison, whereas the senior coder coded it as a similarity comparison, arguing that a similarity is being drawn between two states of inability.

*Global vs. specific.* In the reliability sample for children, there were 5 total disagreements on whether a comparison was global or specific. Most of these were driven by the senior coder being more conservative, coding comparisons as global unless a feature was unambiguously specified in the child’s language. For example, ‘doesn’t look like a J’ was coded by the more junior coder as specifying Shape. The more senior coder coded this as a global comparison, since the child’s language did not specify that the target was the same shape as a J. In the reliability sample for parents, there were 39 total disagreements on whether a comparison was global or specific. Most of these proceeded from the more junior coder not incorporating features that were specified in the preceding or the following utterance. As stated in the coding scheme in Text S1 above, we aimed to use the transcript context and the video if necessary to determine in full what was being compared. For example, the utterance ‘just like our baby’ was coded as a specific comparison with the feature Shape because the previous utterance was ‘I was little’.

*Feature category/feature type.* These disagreements tended to occur in cases of infrequently specified features, such as comparing the dryness of two marker pens, where coders disagreed on whether this feature was Sensory or Evaluative (resolving on Sensory). Other disagreements came from the more junior coder coding the feature that was implied, rather than the feature that was stated in the language (e.g., Preference where the word used was ‘better’, Age where the word used was ‘bigger’).

*Within- or between-category.* This was the most difficult category to code reliably, since it involved placing naturalistically varying objects into a limited set of superordinate categories. Subjective variation in these judgements inevitably occurred. For example, ‘your boat’s kind of like a raft’ was coded as between-category by the more junior coder, but as within-category by the more senior coder; ‘you’re throwing more on the floor than you’re getting in the jar’ was coded as within-category by the more junior coder, but as between-category by the more senior coder. Future work should consider whether a better categorisation scheme could be developed to capture this phenomenon.

**Text S3**. Missing data and imputation.

We had some missing values in our data set. These were dealt with as described in our preregistration ((<https://osf.io/gaq97>). We reproduce that description here. Imputed data were used for all analyses involving missing values: namely, the confirmatory analyses for the responsiveness hypothesis, and the exploratory analyses involving parent responsiveness.

3 data points were missing in total. Firstly, one child never produced a specific comparison, so no parent response to specific comparisons was observable. This meant we had one missing data point for the parent responsiveness predictor, and one missing data point for the parent responsiveness outcome (log child specific comparisons in the two sessions following first parent response). Secondly, one child did not produce their first specific comparison until session 11 of 12, meaning 2 sessions following first parent response were not available. This meant we had one additional missing data point for the parent responsiveness outcome.

We dealt with these missing data points via multiple imputation using the mice library in R (van Buuren & Groothuis-Oudshoorn, 2011) to make full use of the data we had and avoid introducing bias by simply dropping incomplete cases. All variables in the dataset with correlations more than .1 with the incomplete variables were used as predictors for imputation. In addition, three auxiliary variables were included as predictors for imputation. 1) For one of the two children whose parent responsiveness outcome was missing, specific comparisons produced during one session after onset were available. Since this correlated across children .40 with the parent responsiveness outcome, we used this variable as a predictor for imputing this child’s outcome. 2) For imputing parent responsiveness, we included an analogous measure: parent mean length of utterance during the session in which their child began producing multi-clause utterances. This correlated .32 with parent responsiveness to children’s specific comparisons, suggesting it was a reasonably related behaviour. 3) For imputing the parent responsiveness outcome, we included an analogous measure of child production: cumulative word types produced by the child over sessions 1-12 (from 14 to 58 months). This correlated .25 with the parent responsiveness outcome.

For the outcome models that used imputed data, each model was run on 5 multiply imputed datasets. Estimates and standard errors were pooled according to Rubin’s rules (Rubin, 1987).

**Text S4.** Aim 2 analysis using untransformed original counts (replicating Silvey et al., 2017).

As noted in the text, the analyses on the smaller dataset reported in Silvey et al. (2017), which the Aim 2 analyses in the current paper replicate and extend, used untransformed comparison counts. In our analyses, we use log-transformed comparison counts, since the data are notably skewed. However, to check whether the original results replicate, we also report here analyses on the larger dataset using untransformed counts.

Main analysis

Our main analysis using untransformed counts found that specific comparison count was a significant predictor of score in the Woodcock-Johnson Verbal Analogies: $\beta=0.07, t=2.21,p=.033$. This corresponded to a standardised effect size of 0.33, a small to medium effect. PPVT was justified for inclusion in the model. Adjusted $R^{2}$ for the model was .25, suggesting that together, specific comparisons and PPVT explain around a quarter of the variance in verbal analogy scores. We also found that untransformed specific comparison count was a significant predictor of score in Raven’s Progressive Matrices: $\beta=0.42,t=3.73,p<.001$. This corresponded to a standardised effect size of .51, a medium effect. Adjusted $R^{2}$ for the model was .24, suggesting that this predictor explains around a quarter of the variance in non-verbal analogy scores. PPVT was not justified for inclusion in this model. Figure S1 shows the scatterplots of the relationships between specific comparisons and the two outcomes.


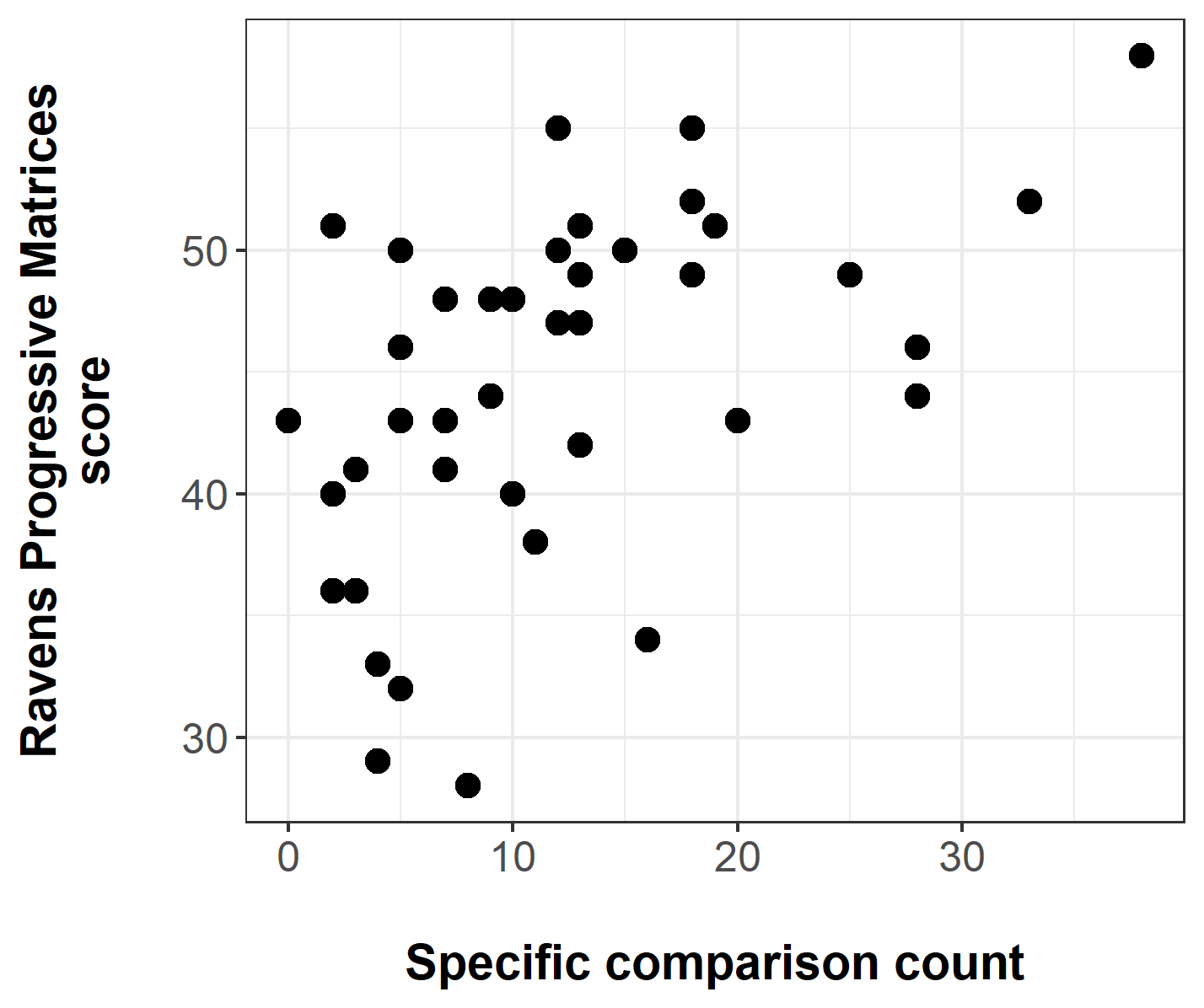


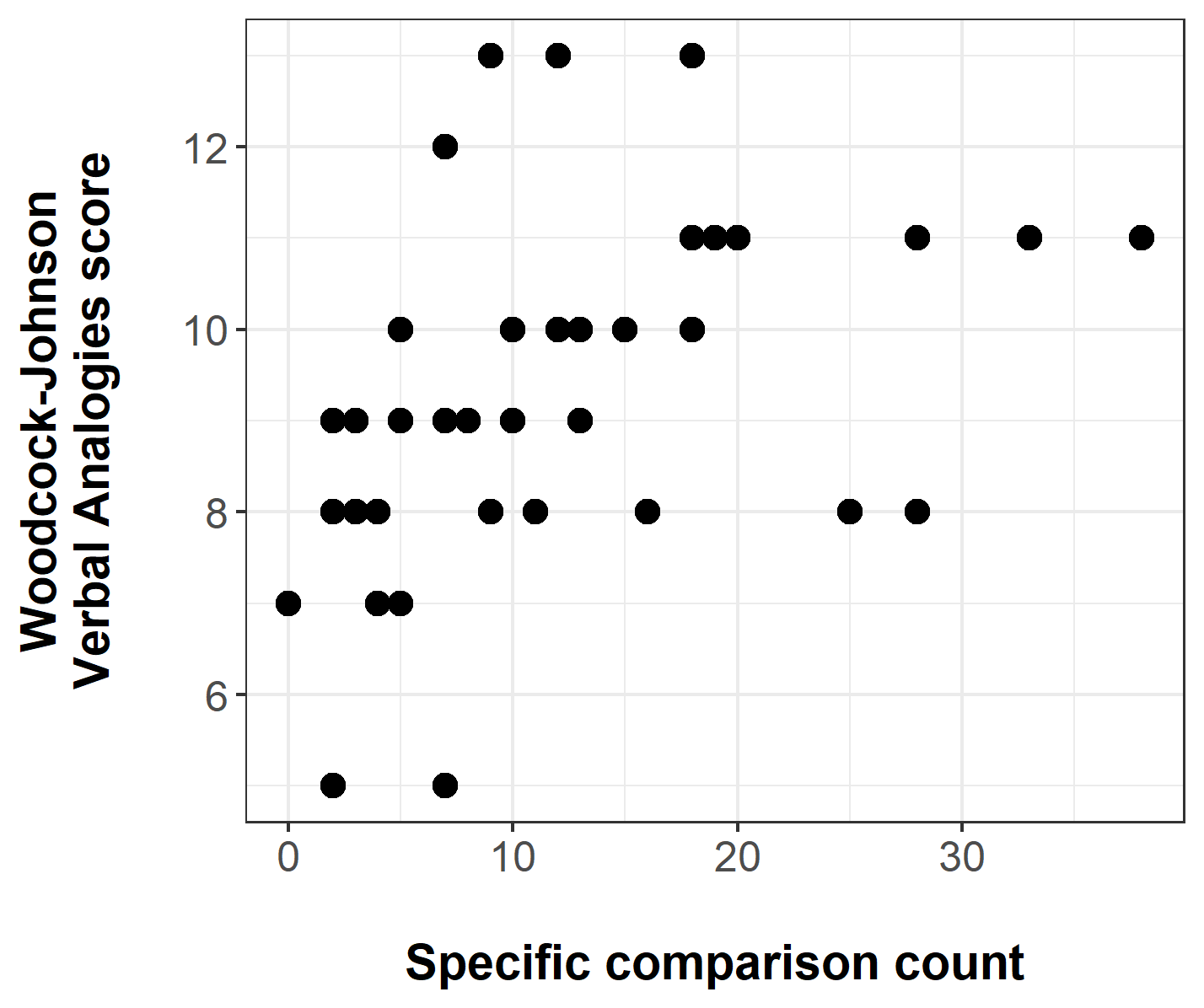


Figure S1. Scatterplots showing the relationship between specific comparison count (x axis) and analogical reasoning outcomes. Left panel shows score in Woodcock-Johnson Verbal Analogies (y axis) and right panel shows score in Raven’s Progressive Matrices (y axis).

Results from the model for Woodcock-Johnson Verbal Analogies are shown in Table S1, and for Raven’s Progressive Matrices in Table S2.

Table S1. Results from statistical model analysing the effect of specific comparison count on child score in Woodcock-Johnson Verbal Analogies, controlling for PPVT score at 54 months.

| **Predictor** | **b** | **β** | **SE** | **t** | **p** |
| --- | --- | --- | --- | --- | --- |
| Specific comparison count  PPVT | 0.07  0.03 | 0.33  0.31 | 0.032  0.014 | 2.21  2.10 | .033  .043 |

Table S2. Results from statistical model analysing the effect of specific comparison count on child score in Raven’s Progressive Matrices. PPVT score at 54 months was not justified for inclusion as a predictor.

| **Predictor** | **b** | **β** | **SE** | **t** | **p** |
| --- | --- | --- | --- | --- | --- |
| Specific comparison count | 0.42 | 0.51 | 0.113 | 3.73 | <.001 |

**Text S5.** Additional exploratory analyses

Spatial comparisons and child gender

Many of the specific comparisons produced by both children and their parents are spatial, discussing features such as size, height and speed. Parents may not use as much spatial language with girls as with boys (Levine, Ratliff, Huttenlocher, & Cannon, 2012). One natural question is whether this dominance of spatial comparisons affects the prevalence of specific comparisons (either in parents’ input or in children’s own production) asymmetrically for boys and girls.

We can break this question down into the following components. Firstly, do girls produce fewer specific comparisons than boys? If so, is this driven by girls producing fewer spatial comparisons? Secondly, do parents produce fewer specific comparisons in input to girls? If so, is this driven by parents of girls producing fewer spatial comparisons?

To address the first question: girls produced fewer specific comparisons on average than boys, but this difference was not statistically significant: $b=-0.32,t=-1.36,p=.181$. Figure S2 shows the proportion of children’s specific comparisons that were spatial for boys and girls. While more girls are clustered in the middle of the range, overall the distributions are similar, with girls averaging 43% spatial comparisons and boys averaging 45%. These proportions did not significantly differ by gender: *Z* = 0.16, *p* = .869.


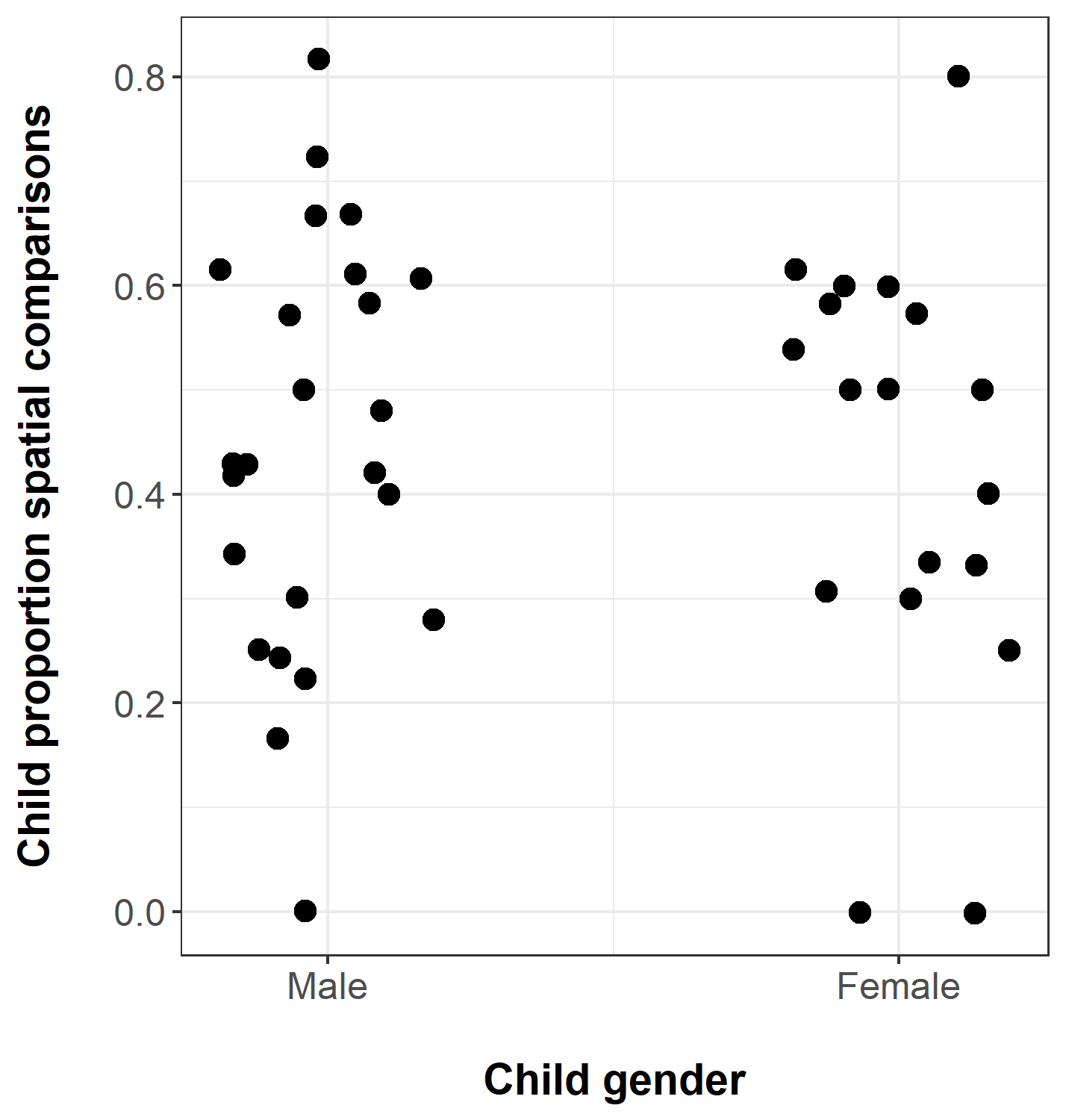


Figure S2. The proportion of specific comparisons produced by male and female children that specified a spatial feature (e.g., size, height, volume).

To address the second question: child gender was negatively but not significantly related to log specific comparison input from parents during the first three sessions ($b=-0.21,t=-0.90,p=.372$). As was the case for children, the proportion of parents’ specific comparisons that were spatial covered a similar distribution regardless of child gender. Taken together, these results suggest that differences in spatial language addressed to boys and girls do not have a substantial impact on parents’ specific comparison input or children’s production of specific comparisons.

Parents’ comparisons before and after child onset

In our main analyses, we found that children made several ordering distinctions between types of comparison. Most children produced global comparisons before specific comparisons, similarity comparisons before difference comparisons, and within-category comparisons before between-category comparisons. By contrast, we found that parents did not generally make an ordering distinction between global and specific comparisons, or between similarity and difference comparisons; around half of parents produced within-category comparisons before between-category comparisons, whereas the other half of parents produced them at the same time.

What this analysis did not investigate is whether parents’ comparisons changed after their child started producing comparisons themselves. In particular, did parents produce more comparisons after child onset, and did they produce a higher proportion of the more sophisticated kinds of comparison: specific rather than global, difference rather than similarity, and between-category rather than within-category?

To investigate this question, we divided each parent’s comparisons into a) before onset, meaning all comparisons produced before the child’s onset session, and b) after onset, meaning all comparisons produced during and after the child’s onset session. We found the following:

Parents produced more comparisons per session after child onset (Figure S3). A model predicting log-transformed average comparisons per session from time (before or after) found that this increase was significant: $b=0.53,t=3.70,p<.001$.


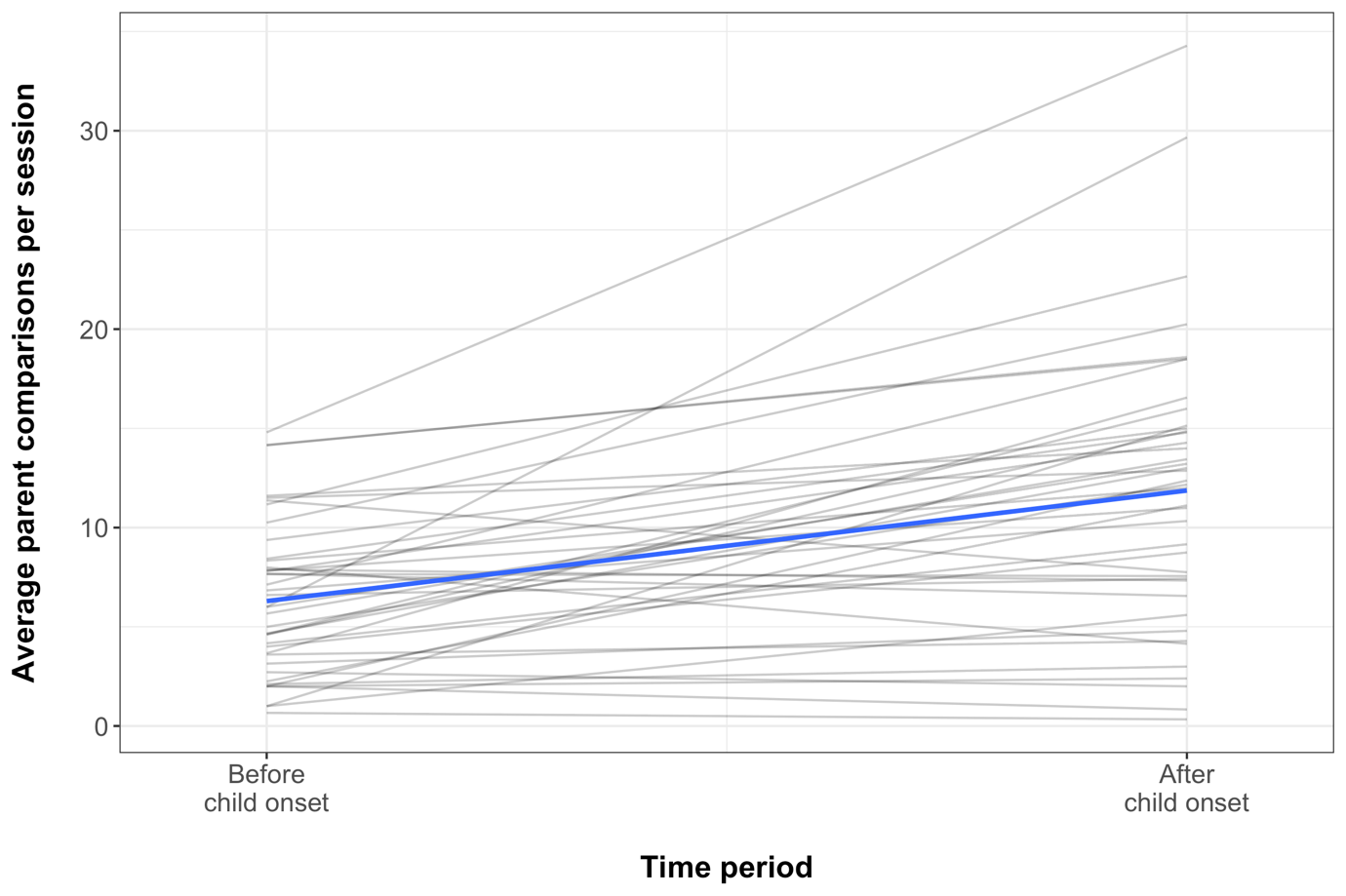


Figure S3. Average number of comparisons produced by parents before and after their child’s first comparison. Grey lines show individual parents, blue line the average trajectory.

Parents overall produced a higher proportion of specific comparisons after child onset than before: $b=0.31,z=3.57,p<.001$. As Figure S4 shows, there was a large amount of variation between parents. Parents overall produced a higher proportion of difference comparisons after their child’s first comparison than before: $b=0.51,z=5.16,p<.001$ (Figure S5). However, the proportion of between-category comparisons was not significantly different before and after child onset: $b=-0.15,z=-1.35,p=.178$.

For all these changes in parents’ comparisons, it is important to note that we cannot be sure they happened *because* of child onset; we may instead have captured a change over time that would have happened in any case as children got older.


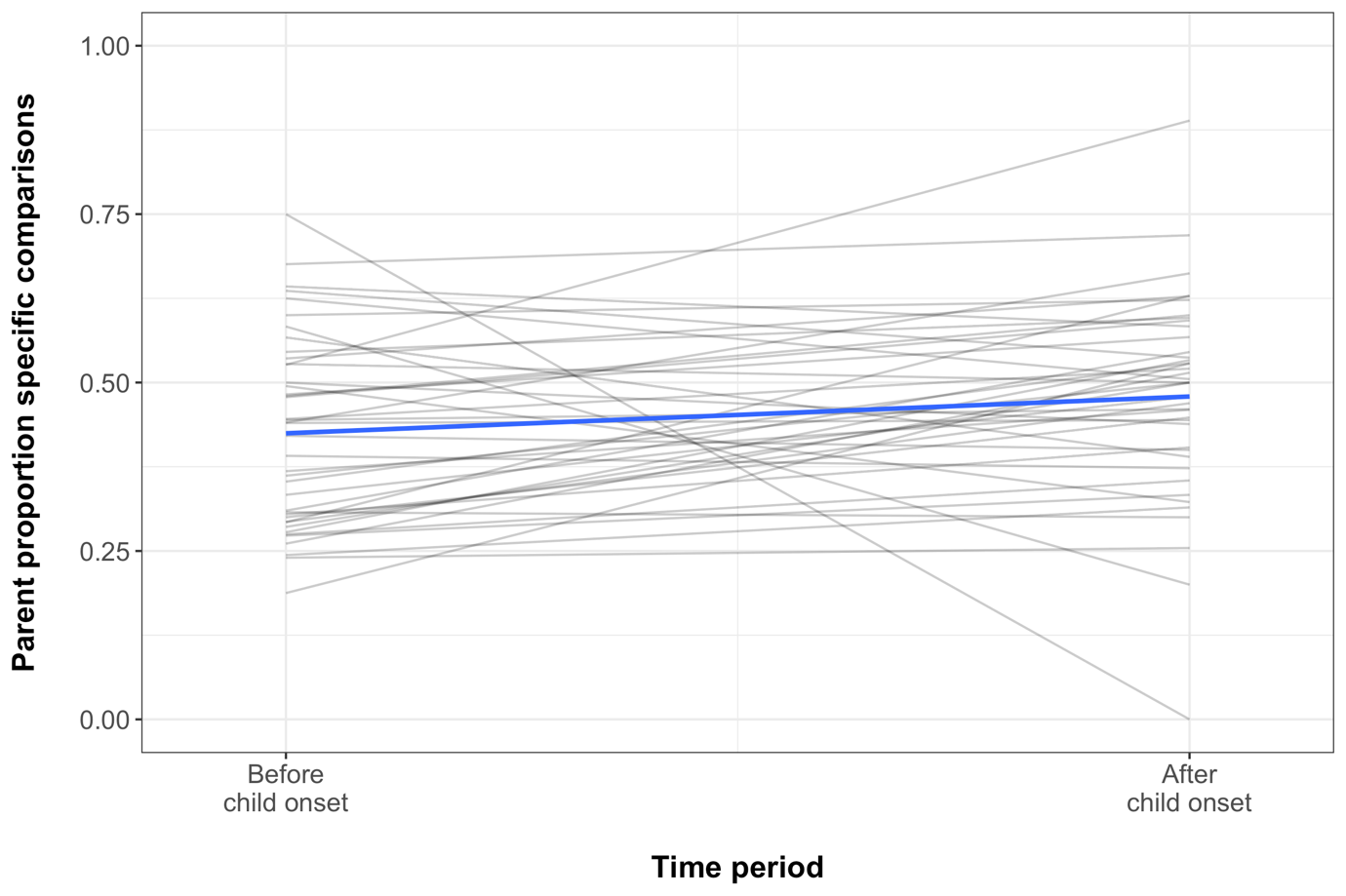


Figure S4. Average proportion of comparisons produced by parents that were specific, before and after their child’s first comparison. Grey lines show individual parents, blue line the average trajectory.


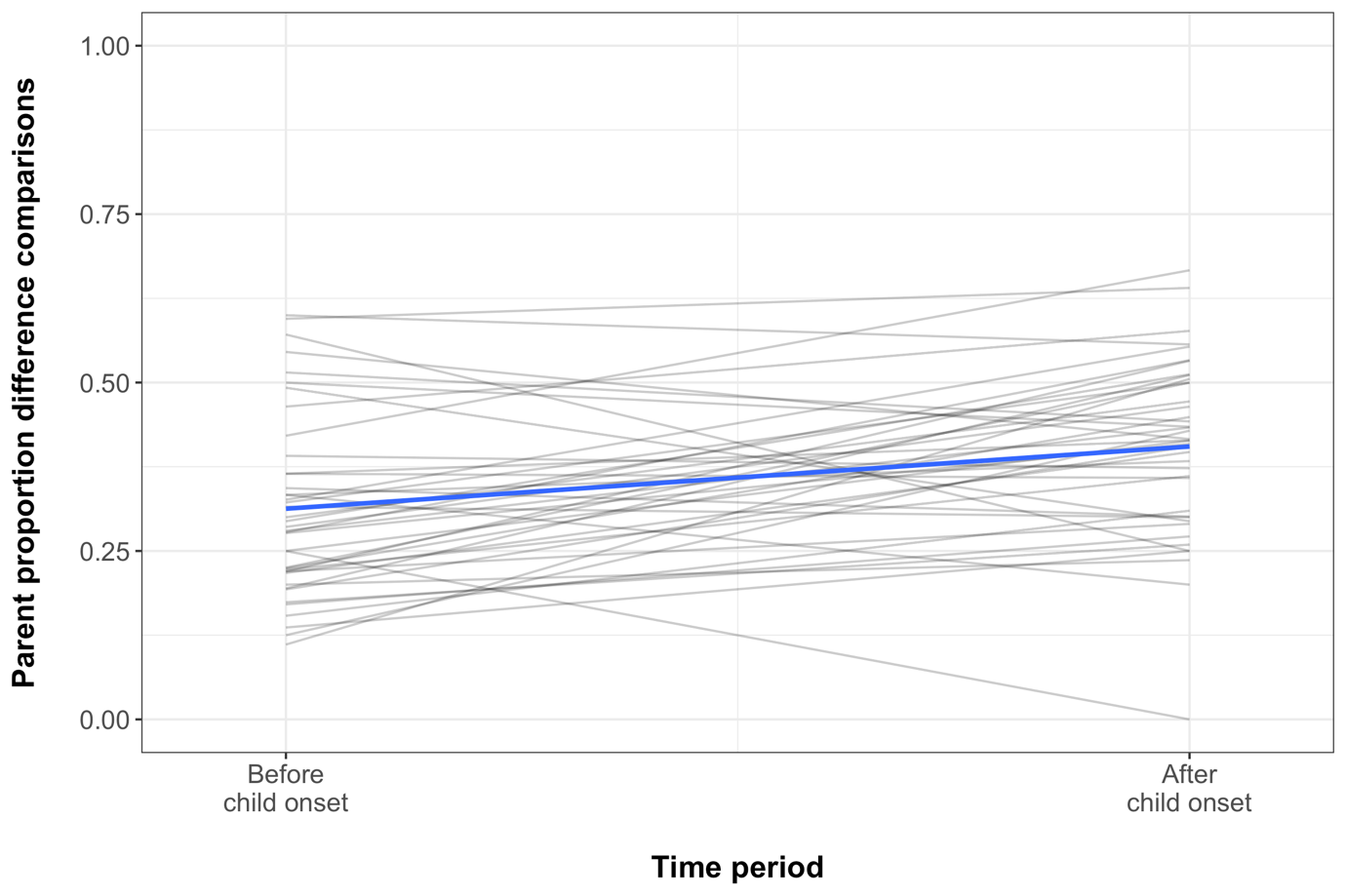


Figure S5. Average proportion of comparisons produced by parents that highlighted a difference, before and after their child’s first comparison. Grey lines show individual parents, blue line the average trajectory.

Children’s earliest comparisons: differences based on age of onset

The children in our sample varied widely in the age at which they first began producing comparisons. The earliest age we observed was 26 months, and the latest 58 months. These children were at very different developmental stages both cognitively and with respect to their language. We therefore wanted to ask whether the first comparisons children produced differed systematically for early versus late starters. There are two broad possibilities: 1) early-emerging comparisons were less sophisticated than later-emerging comparisons in terms of the types of comparisons they included; 2) children’s first comparisons looked broadly the same regardless of when they emerged. To investigate this question, we contrasted the first comparisons produced by early starters (who produced their first comparison in session 4 or 5, at age 26-30 months) and by late starters (who produced their first comparison in session 7 or later, at age 38 months or older). Here, ‘first comparisons’ refers to the comparisons the child produced during the first session in which they produced any. 15 children were classified as early starters, and 15 as late starters. We then compared these groups in terms of the prevalence of more sophisticated types of comparison: specific comparisons, difference comparisons, and between-category comparisons. Early starters and late starters looked similar in terms of specific comparisons: in both groups, 9 out of 15 children did not produce specific comparisons during their first session, whereas 6 did. They also looked similar in terms of difference comparisons: in the early starters group, 10 out of 15 did not produce difference comparisons during their first session, whereas 5 did; in the late starters group, 12 out of 15 did not produce difference comparisons during their first session, whereas 3 did. One potential difference could be seen in between-category comparisons: while only 3 out of 15 early starters made a between-category comparison during their first session, 8 out of 15 late starters did. In general, then, children’s first comparisons looked similar regardless of when they emerged: they were more likely to be global than specific, and more likely to point out similarity than difference. However, older children may have found it easier to make comparisons between objects from different categories from the start.

Children’s comparisons: change over time

While the descriptive analyses reported in the main analyses described ordering trends in particular types of comparison, they did not investigate whether the prevalence of the different comparison types in children’s speech changed from early to later in their development. To answer this question, we took a similar approach to the one we used to examine parents’ comparisons before and after child onset. For each child, we classed as ‘early’ the comparisons they produced in the first session during which they produced a comparison and the two sessions immediately following. We classed as ‘late’ the comparisons they produced in the three sessions immediately following the early sessions. For a child who began producing comparisons in session 4, ‘early’ comparisons would be those produced in sessions 4, 5, and 6; ‘late’ comparisons would be those produced in sessions 7, 8, and 9. For this analysis, we had to exclude the 5 children who began producing comparisons in session 8 or later, since they would not have enough ‘late’ sessions to compare with the other children. We then investigated whether children’s late sessions included more comparisons, and whether a higher proportion of these late comparisons were specific rather than global, highlighted difference rather than similarity, and were between-category rather than within-category.

Children’s late sessions contained more comparisons than their early sessions on average: $b=0.37,t=3.50,p<.001$ (Figure S6). Children did not produce a significantly higher proportion of specific comparisons late than early: $b=0.09,z=0.35,p=.728$; they also did not produce a significantly higher proportion of between-category comparisons late than early: $b=-0.20,z=-0.81,p=.420$. However, children did produce a significantly higher proportion of difference comparisons late than early: $b=0.55,z=2.57,p=.010$ (Figure S7).


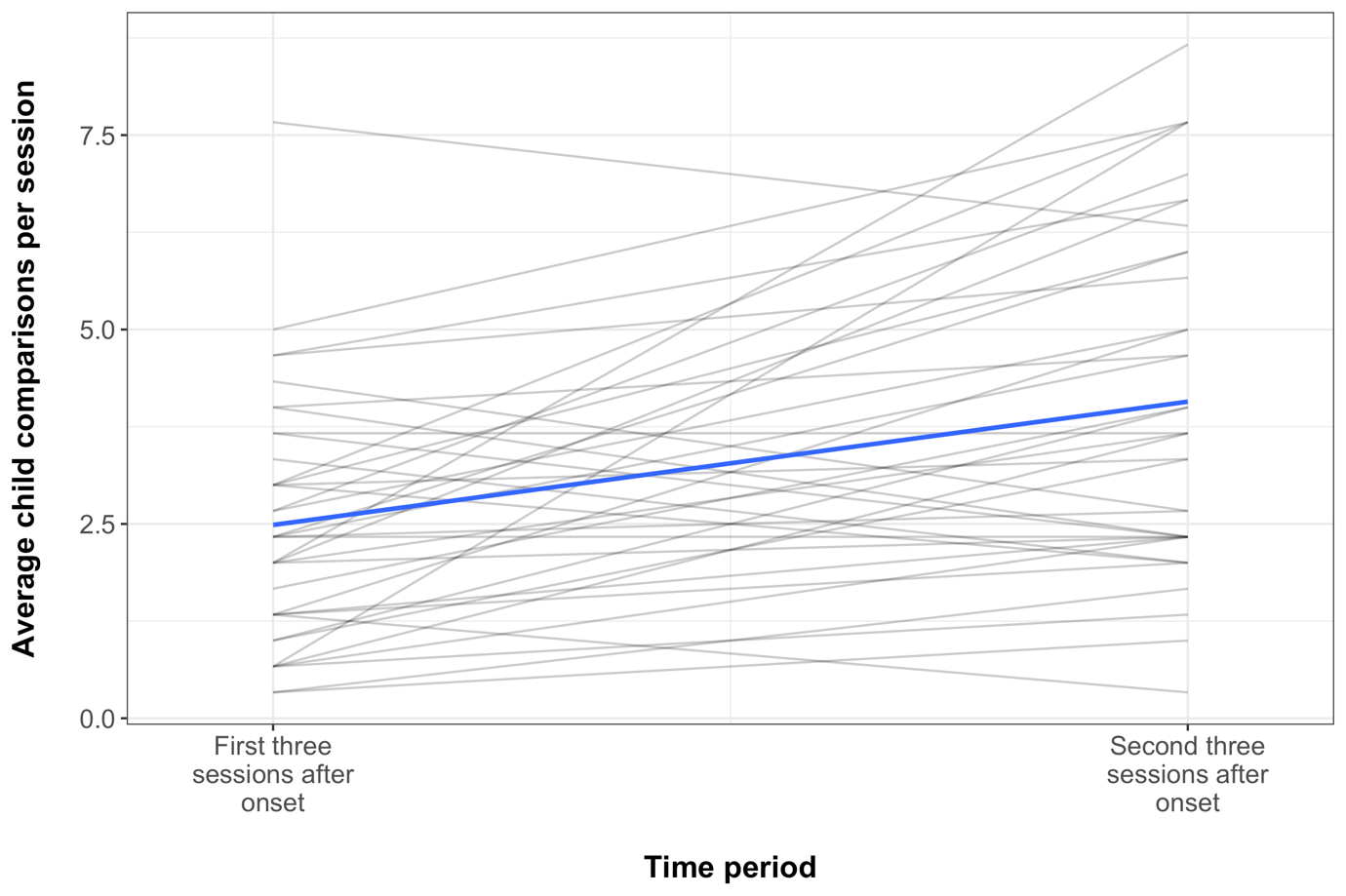


Figure S6. Average number of comparisons produced by children in the first three sessions during which they produced comparisons (early), and the three sessions after that (late). Grey lines show individual children, blue line the average trajectory.


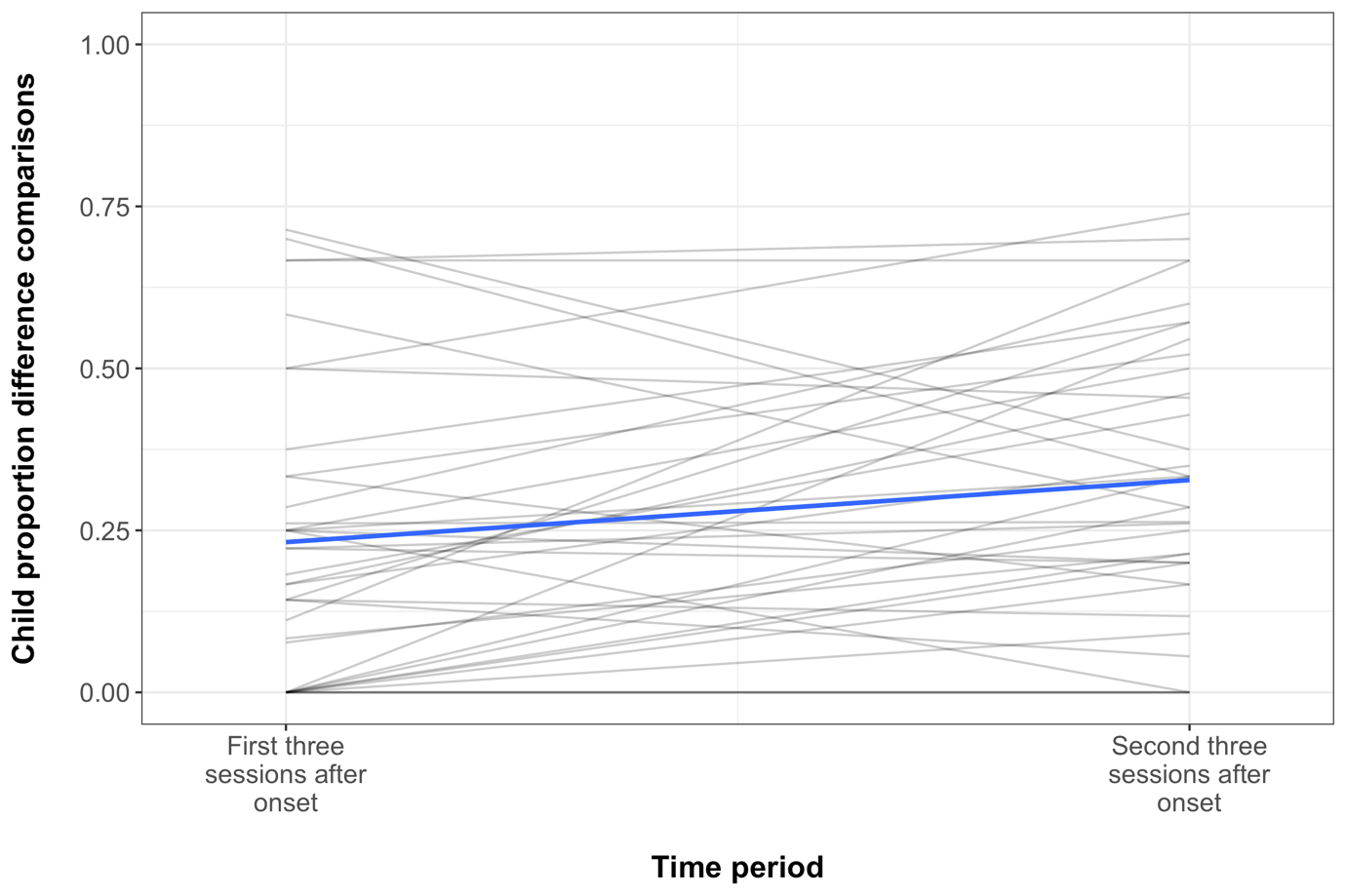


Figure S7. Average proportion of comparisons produced by children that highlighted a difference, in the first three sessions during which they produced comparisons (early) and the following three sessions (late). Grey lines show individual children, blue line the average trajectory.

**References**

Levine, S. C., Ratliff, K. R., Huttenlocher, J., & Cannon, J. (2012). Early puzzle play: A predictor of preschoolers’ spatial transformation skill. *Developmental Psychology*, *48*(2), 530–542. https://doi.org/10.1037/a0025913

Özçalışkan, Ş., Goldin-Meadow, S., Gentner, D., & Mylander, C. (2009). Does language about similarity play a role in fostering similarity comparison in children? *Cognition*, *112*(2), 217–228. <https://doi.org/10.1016/j.cognition.2009.05.010>

Rubin, D. B. (1987). *Multiple imputation for nonresponse in surveys*. London: John Wiley & Sons.

Silvey, C., Gentner, D., Richland, L. E., & Goldin-Meadow, S. (2017). Children’s spontaneous comparisons from 26 to 58 months predict performance in verbal and non-verbal analogy tests in 6th grade. In G. Gunzelmann, A. Howes, T. Tenbrink, & E. Davelaar (Eds.), *Proceedings of the 39th Annual Meeting of the Cognitive Science Society* (pp. 1072–1077). Austin, TX: Cognitive Science Society.
